# Crystal structures of two dimeric nickel di­phenyl­acetate com­plexes

**DOI:** 10.1107/S2056989019014063

**Published:** 2019-10-29

**Authors:** A. A. Nikiforov, D. O. Blinou, E. N. Dubrov, N. S. Panina, A. I. Ponyaev, V. V. Gurzhiy, A. V. Eremin, A. I. Fischer

**Affiliations:** a St Petersburg State Institute of Technology, Moskovsky pr. 26, 190013 St Petersburg, Russian Federation; b Institute of Macromolecular Compounds, Russian Academy of Sciences, Bolshoy pr. 31, 199004 St Petersburg, Russian Federation; c St Petersburg State University, University Emb. 7/9, 199034, St Petersburg, Russian Federation; d Peter the Great St Petersburg Polytechnic University, Polytechnicheskaya 29, 195251 St Petersburg, Russian Federation

**Keywords:** crystal structure, Ni^II^ dimer, carboxyl­ate com­plex, Hirshfeld surface analysis, hydrogen bonds, π-stacking

## Abstract

In the crystal structures of the title com­pounds, namely μ-aqua-κ^2^ *O*:*O*-di-μ-di­phenyl­acetato-κ^4^ *O*:*O*′-bis­[(di­phenyl­acetato-κ*O*)bis­(pyridine-κ*N*)nickel(II)], [Ni_2_(C_14_H_11_O_2_)_4_(C_5_H_5_N)_4_(H_2_O)] (**1**) and μ-aqua-κ^2^ *O*:*O*-di-μ-di­phenyl­acetato-κ^4^ *O*:*O*′-bis­[(2,2′-bi­pyridine-κ^2^ *N*,*N*′)(di­phenyl­acetato-κ*O*)nickel(II)]–aceto­nitrile–di­phenyl­acetic acid (1/2.5/1), [Ni_2_(C_14_H_11_O_2_)_4_(C_10_H_8_N_2_)_2_(H_2_O)]·2.5CH_3_CN·C_14_H_12_O_2_ (**2**), the com­plex units are stabilized by a variety of intra- and inter­molecular hydrogen bonds, as well as C—H⋯π and π–π contacts between the aromatic systems of the pyridine, dipyridyl and di­phenyl­acetate ligands. Despite the fact that the di­phenyl­acetate ligand is sterically bulky, this does not inter­fere with the formation of the described aqua-bridged dimeric core, even with a 2,2′-bi­pyridine ligand, which has a strong chelating effect.

## Abstract 2

The mol­ecular and crystal structures of μ-aqua-κ^2^ *O*:*O*-di-μ-di­phenyl­acetato-κ^4^ *O*:*O*′-bis­[(di­phenyl­acetato-κ*O*)bis­(pyridine-κ*N*)nickel(II)] and μ-aqua-κ^2^ *O*:*O*-di-μ-di­phenyl­acetato-κ^4^ *O*:*O*′-bis­[(2,2′-bi­pyridine-κ^2^ *N*,*N*′)(di­phenyl­acetato-κ*O*)nickel(II)]–aceto­nitrile–di­phenyl­acetic acid (1/2.5/1) are reported. Hirshfeld surface analysis of both com­pounds have been carried out.

## Chemical context   

The title com­pounds, **1** and **2**, were synthesized as a part of our ongoing research on catalytically active polynuclear Ni^II^ and Co^II^ carboxyl­ate com­plexes with various structures and nuclearity in lactone ring-opening polymerization and ketone hydro­silylation. They belong to the type of aqua-bridged dinickel(II) carboxyl­ates with the general formula [*M* ^II^_2_(μ-H_2_O)(μ-O_2_C*R*)_2_(O_2_C*R*)_2_ *L_n_*] (*n* = 4 in the case of a monodentate ligand or 2 in the case of bidentate coordination), well known since the 1970s (Turpeinen, 1976 ▸). In this work, it is shown that the reaction of highly reactive synthetic hellyerite, NiCO_3_·5.5H_2_O (Bette *et al.*, 2016 ▸), a stoichiometric amount of di­phenyl­acetic acid and treatment with the *N*-donor ligand leads to self-assembly of the title com­pounds. The use of sterically bulky ligands and ligands that are prone to the formation of multiple intra- and inter­molecular inter­actions can give unexpected and inter­esting results (Lee *et al.*, 2002 ▸; Nikolaevskii *et al.*, 2016 ▸).

## Structural commentary   

**Figure 1:**
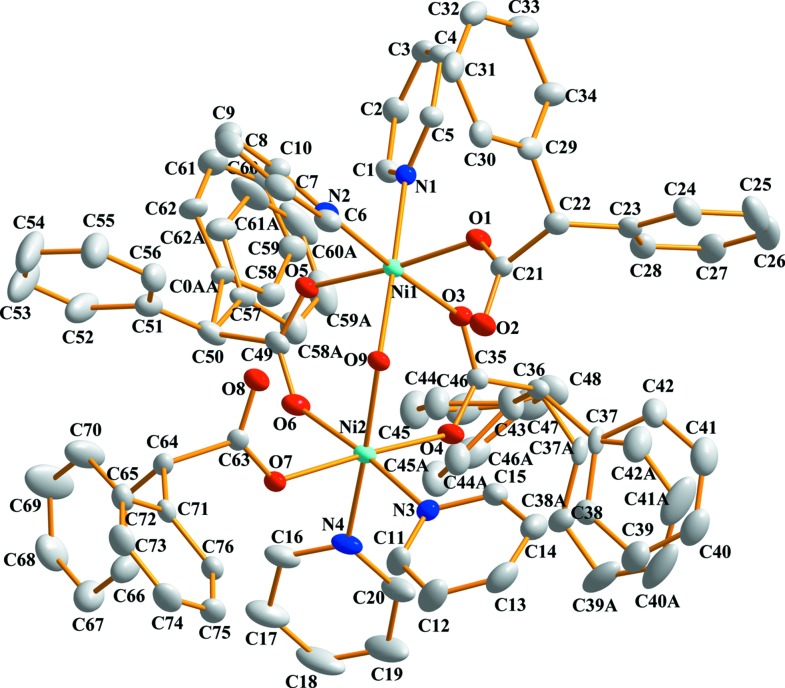
The mol­ecular structure of **1**, showing the atom-labelling scheme. Displacement ellipsoids are drawn at the 50% probability level. H atoms have been omitted for clarity.

In the title binuclear com­plexes, **1** and **2**, each Ni^II^ ion is six-coordinated by two carboxyl­ate O atoms from two bidentate-bridged di­phenyl­acetate ligands, one O atom from a monodentate di­phenyl­acetate ligand, two N atoms from two pyridine (Py) (for **1**) or one 2,2′-bi­pyridine (Bipy) ligand (for **2**) and one O atom from a bridging aqua ligand in an octa­hedral geometry (Figs. 1 ▸ and 2 ▸). The com­plexes display idealized twofold symmetry, with the axis passing through the bridging water mol­ecule. The Ni⋯Ni distances in the com­plexes are 3.5779 (4) (for **1**) and 3.4826 (5) Å (for **2**). Each monodentate coordinated di­phenyl­acetate ligand is involved in the formation of an intra­molecular hydrogen bond with a bridging water mol­ecule. Hydrogen-bond geometries are specified in Tables 1 ▸ and 2 ▸.
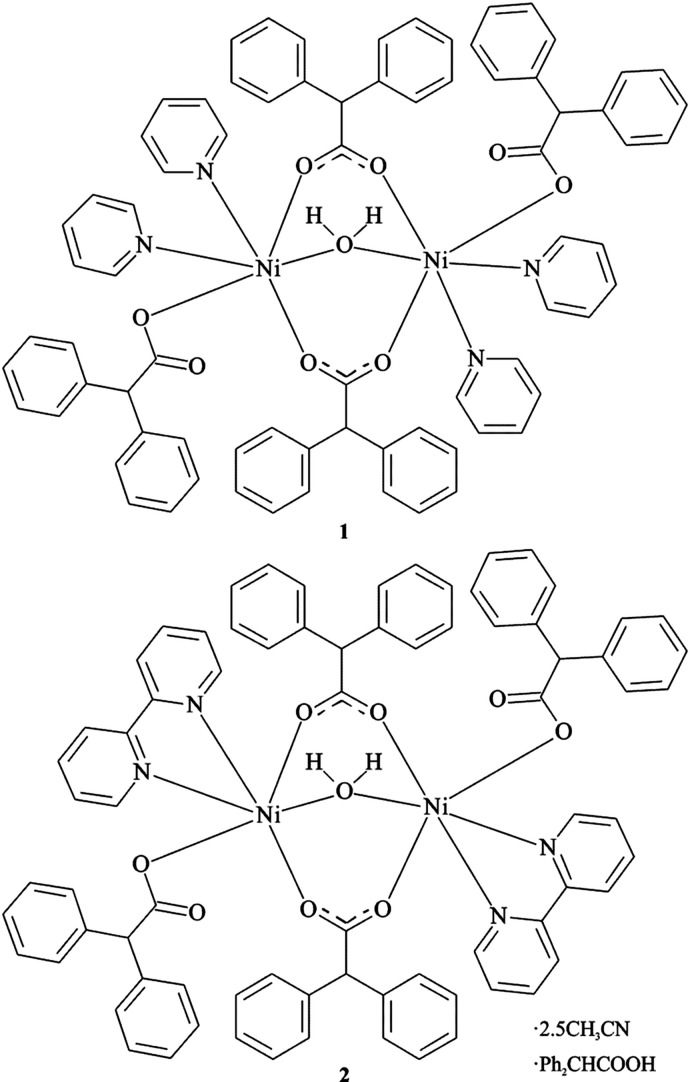


## Supra­molecular features   

In the crystal packing of com­pound **1**, mol­ecules are combined into pairs connected by a centre of symmetry using offset face-to-face π–π stacking inter­actions, in which coordinated pyridine ligands of each com­plex [the N1/C1–C5⋯N1^iii^/C1^iii^–C5^iii^ plane-to-plane distance = 3.342 Å; symmetry code: (iii) −*x* + 2, −*y* + 1, −*z*] are involved. These pairs, which result from the intermolecular hydrogen bonds between Py ligands and O atoms of carbonyl groups of di­phenyl­acetate ligands [C8—H8⋯O8^i^ and C12—H12⋯O2^ii^; symmetry codes: (i) −*x* + 1, −*y* + 1, −*z* + 1; (ii) −*x* + 1, −*y* + 1, −*z* + 1], form layered structures parallel to the (101) plane. The layers form a 3D supra­molecular structure through inter­molecular C—H⋯π contacts, as well as π–π stacking inter­actions between the phenyl substituents of di­phenyl­acetate ligands [C57—C62⋯*Cg*1^iv^, plane-to-centroid distance = 3.231 Å; *Cg*1 is the centroid of the C57*A*–C62*A* ring; symmetry code: (iv) −*x* + 2, −*y* + 2, −*z*].

In the crystal structure of com­pound **2**, each mol­ecule of the com­plex is associated with two neighbouring mol­ecules *via* π–π stacking inter­actions between Bipy ligands [N1—N2⋯N1^i^—N2^i^ and N3—N4⋯N3^ii^—N4^ii^, plane-to-plane distances = 3.342 (2) and 3.310 (1) Å, respectively; symmetry codes: (i) −*x* + 1, −*y* + 1, −*z* + 2; (ii) −*x* + 1, −*y* + 2, −*z* + 1]. As a result, polymer chains are formed along the [01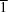] direction, in which every pair of neighbouring mol­ecules is connected by a centre of symmetry. The chains are linked through C—H⋯π inter­actions of phenyl substituents of di­phenyl­acetate ligands and thereby form a three-dimensional supra­molecular framework. Aceto­nitrile solvent mol­ecules are associated with com­plex units *via* van der Waals inter­actions.

## Hirshfeld surface analysis   

**Figure 3:**
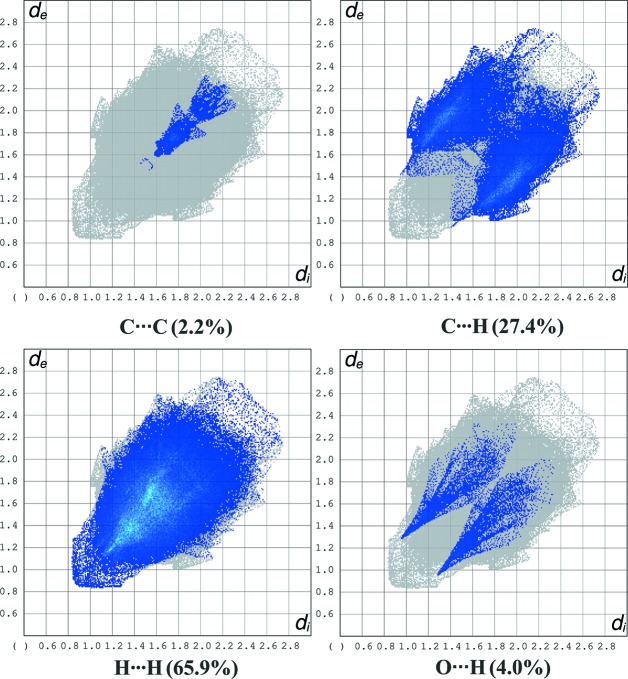
The two-dimensional fingerprint plots for com­pound **1**.

In order to visualize and qu­anti­tatively describe inter­molecular inter­actions in the crystal packing of com­plexes **1** and **2**, Hirshfeld surface analysis (Spackman & Jayatilaka, 2009 ▸) and two-dimensional fingerprint plots (McKinnon *et al.*, 2007 ▸) were carried out and generated using *CrystalExplorer* (Version 17; Turner *et al.*, 2017 ▸). The percentage contributions of the inter­molecular inter­actions to the Hirshfeld surface are shown in Figs. 3 ▸ (for **1**) and 4 ▸ (for **2**).

**Figure 5:**
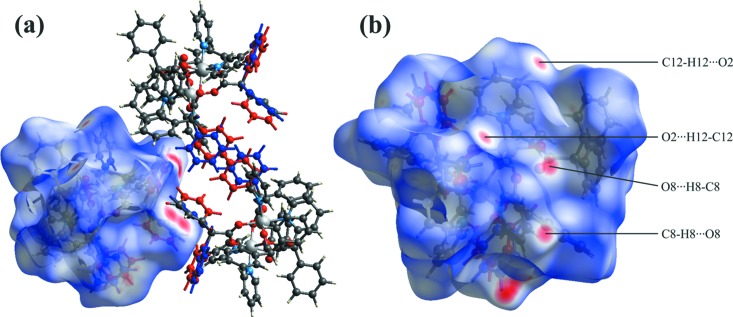
The Hirshfeld surface mapped over *d*_norm_ for com­pound **1** in the range −0.4078 to 1.4837 a.u.

For com­pound **1**, the largest bright-red spots on the Hirshfeld surface near the phenyl substituents (C37–C42, C37*A*–C42*A* and C43–C48) of the di­phenyl­acetate ligands (Fig. 5 ▸ *a*) are indicative of C—H⋯π inter­actions. Such spots are associated with disorder of the aforementioned phenyl substituents. In addition, other bright-red zones correspond to the weak hydrogen bonds C8—H8⋯O8^ii^ and C12—H12⋯O2^iii^ (Fig. 5 ▸ *b* and Table 1 ▸).

**Figure 6:**
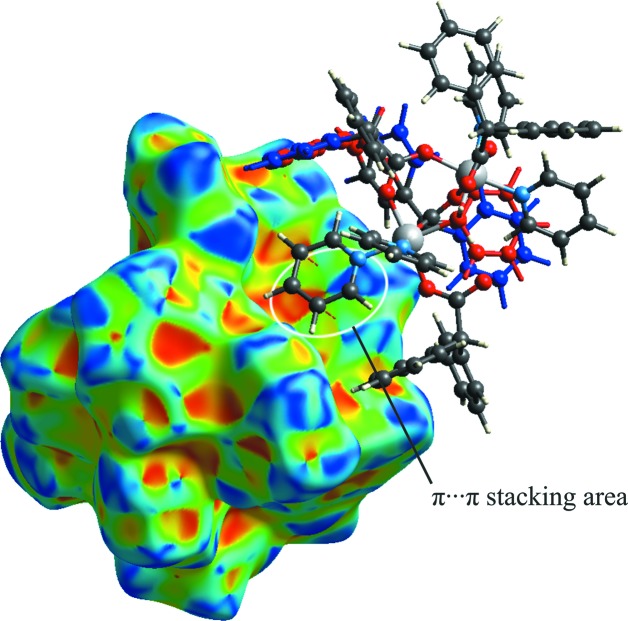
Hirshfeld surface of com­pound **1** plotted over shape-index.

On the surface over shape index (Fig. 6 ▸), areas highlighted by white ellipses and blue and red triangles united along a common vertex are observed, which confirms the presence of close C⋯C inter­planar contacts and, therefore, π–π stacking inter­actions between Py ligands.

**Figure 7:**
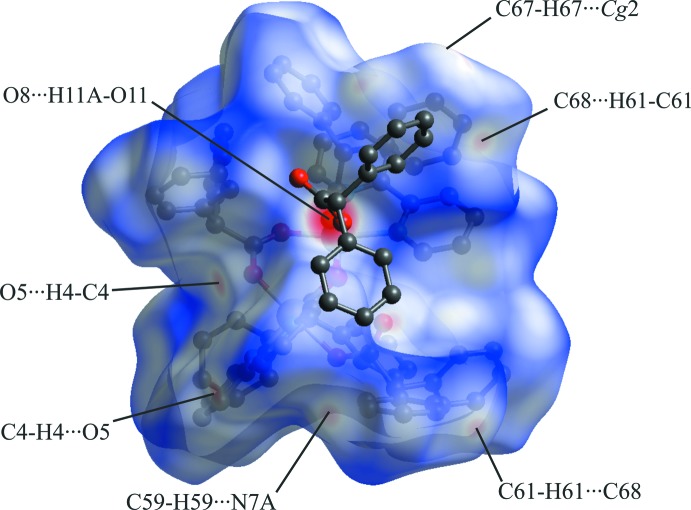
The Hirshfeld surface mapped over *d*_norm_ for com­pound **2** in the range −0.7804 to 1.5753 a.u.

For com­pound **2**, the bright-red spots on the Hirshfeld surface correspond to hydrogen bond O11—H11*A*⋯O8 with the di­phenyl­acetic acid mol­ecule, as well as weak C4—H4⋯O5^i^ and C17—H17⋯O4^ii^ hydrogen bonds between the O atoms of bridged di­phenyl­acetate ligands and Bipy ligands (Fig. 7 ▸ and Table 2 ▸). Other observed red spots correspond to C—H⋯π inter­actions between the phenyl substituents of di­phenyl­acetate ligands [C67—H67⋯*Cg*2^iii^ and C61—H61⋯C68^iv^; *Cg*2 is the centroid of the C23–C28 ring; symmetry codes: (iii) *x* + 1, *y*, *z*; (iv) −*x* + 2, −*y* + 1, −*z* + 1], as well as N⋯H inter­actions involving aceto­nitrile solvent mol­ecules [C59—H59⋯N7*A* ^i^; symmetry code: (i) −*x* + 1, −*y* + 1, −*z* + 2].

**Figure 8:**
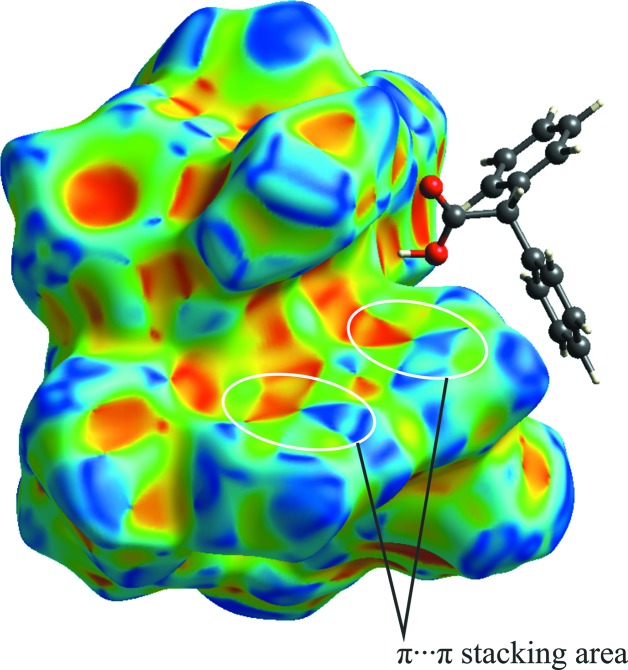
Hirshfeld surface of com­pound **2** plotted over shape-index.

As in the case of com­plex **1**, close C⋯C inter­planar contacts, responsible for π–π stacking inter­actions between Bipy ligands, are displayed as patches of combined blue and red triangles on the surface over shape index (Fig. 8 ▸).

## Database survey   

A search in the Cambridge Structural Database (CSD, Version 5.40, updated February 2019; Groom *et al.*, 2016 ▸) for di­phenyl­acetate com­plexes of transition metals reveals that 54 original structures have been reported. Ten of them are homonuclear com­plexes of Fe, Co and Ni with nuclearity from 3 to 12 and eight of them include the *M*_2_(μ-O_2_CCHPh_2_) fragment and no binuclear structures were found. A survey of the CSD reveals 46 related structures of Ni^II^ carboxyl­ates with a similar structure fragment.

## Synthesis and crystallization   

### Compound 1   

A suspension of synthetic hellyerite, NiCO_3_·5.5H_2_O (0.653 g, 3.0 mmol), in aceto­nitrile (40 ml) was added to a solution of di­phenyl­acetic acid (1.274 g, 6.0 mmol) in 10 ml aceto­nitrile. After full conversion of hellyerite, pyridine (0.485 ml, 6.0 mmol) was added and the solution was refluxed for 15 min. The resulting pale-green–blue solution was cooled to room temperature and filtered. After a few days, blue crystals of **1** were collected by filtration (yield ∼70%).

### Compound 2   

The synthesis of com­pound **2** was carried out in a similar manner to the synthesis of com­pound **1**, but 2,2′-bi­pyridine (0.469 g, 3.0 mmol) was added instead of pyridine. The resulting blue solution was cooled to room temperature and filtered. After a few days, blue crystals of **2** were collected by filtration (yield ∼60%).

## Refinement   

Crystal data, data collection and structure refinement details are summarized in Table 3 ▸. The H atoms of the water mol­ecules were located in difference Fourier maps and refined freely. The other H atoms were placed in calculated positions and refined using a riding model, with C—H = 0.98 Å and *U*_iso_(H) = 1.2*U*_eq_(C) for the tertiary C atoms, C—H = 0.93 Å and *U*_iso_(H) = 1.2*U*_eq_(C) for aromatic C atoms, and C—H = 0.96 Å and *U*_iso_(H) = 1.5*U*_eq_(C) for methyl groups.

## Supplementary Material

Crystal structure: contains datablock(s) global, 1, 2. DOI: 10.1107/S2056989019014063/su5519sup1.cif

Structure factors: contains datablock(s) 1. DOI: 10.1107/S2056989019014063/su55191sup2.hkl

Structure factors: contains datablock(s) 2. DOI: 10.1107/S2056989019014063/su55192sup3.hkl

CCDC references: 1959435, 1959436, 1959435, 1959436

Additional supporting information:  crystallographic information; 3D view; checkCIF report

## Figures and Tables

**Figure 2:**
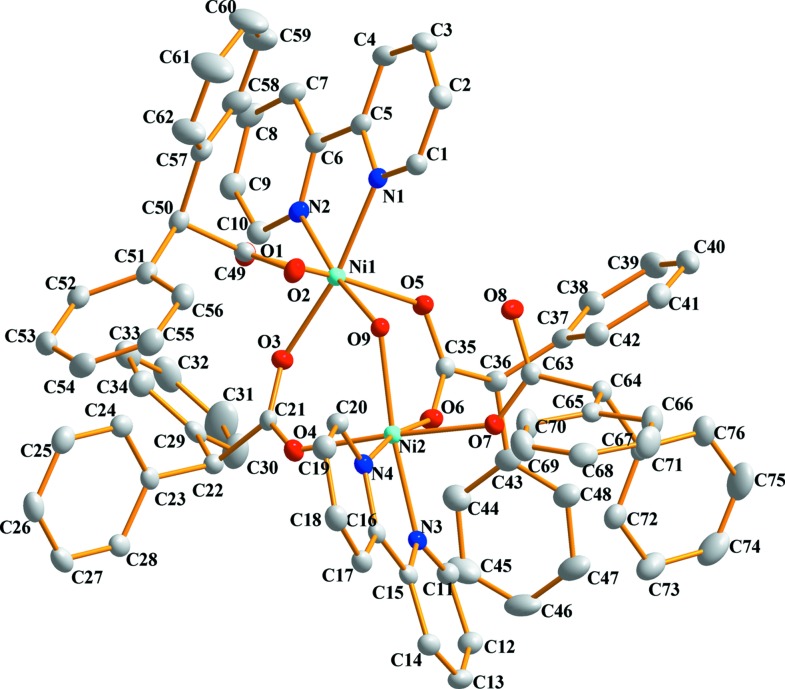
The mol­ecular structure of **2**, showing the atom-labelling scheme. Displacement ellipsoids are drawn at the 50% probability level. H atoms and solvent mol­ecules have been omitted for clarity.

**Figure 4:**
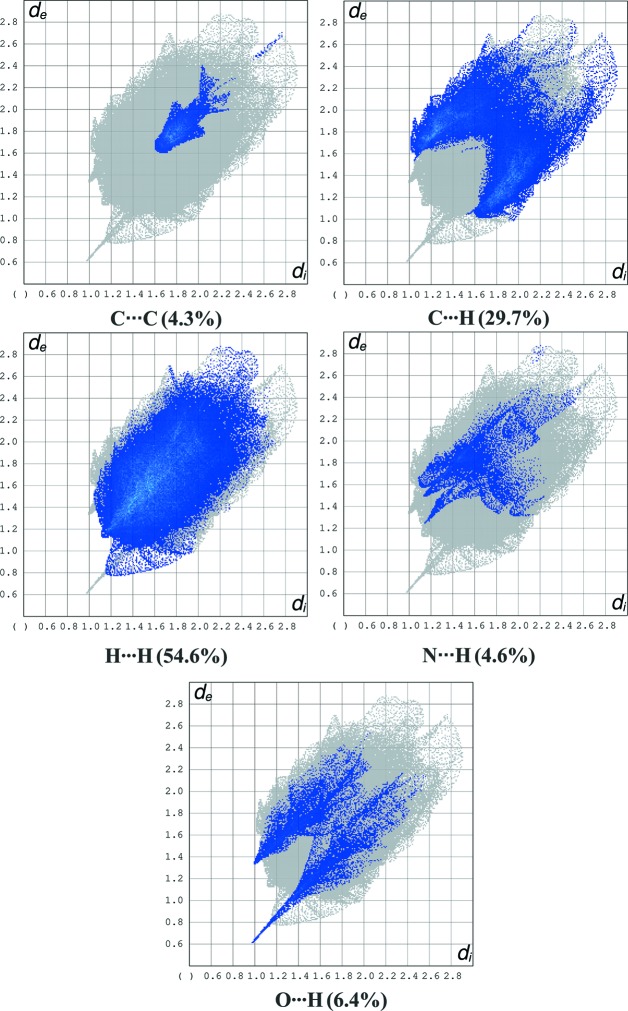
The two-dimensional fingerprint plots for com­pound **2**.

**Table 1:** Hydrogen-bond geometry (Å, °) for **1** [Chem scheme1]

*D*—H⋯*A*	*D*—H	H⋯*A*	*D*⋯*A*	*D*—H⋯*A*
O9—H9*A*⋯O2	0.85 (2)	1.79 (2)	2.6201 (15)	165 (2)
O9—H9*B*⋯O8	0.87 (3)	1.70 (3)	2.5617 (14)	169 (2)
C8—H8⋯O8^i^	0.93	2.39	3.211 (2)	148
C12—H12⋯O2^ii^	0.93	2.46	3.302 (2)	151

Symmetry codes: (i) 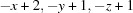; (ii) 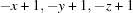.

**Table 2:** Hydrogen-bond geometry (Å, °) for **2** [Chem scheme1]

*D*—H⋯*A*	*D*—H	H⋯*A*	*D*⋯*A*	*D*—H⋯*A*
O9—H9*A*⋯O8	0.91 (3)	1.74 (3)	2.6461 (16)	173 (2)
O9—H9*B*⋯O2	0.96 (3)	1.53 (3)	2.4777 (15)	169 (3)
O11—H11*A*⋯O8	0.82	1.76	2.5599 (16)	164
C4—H4⋯O5^i^	0.93	2.48	3.3253 (19)	152
C17—H17⋯O4^ii^	0.93	2.48	3.3493 (19)	155

Symmetry codes: (i) 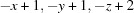; (ii) 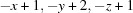.

**Table 3:** Experimental details

	**1**	**2**
Crystal data		
Chemical formula	[Ni_2_(C_14_H_11_O_2_)_4_(C_5_H_5_N)_4_(H_2_O)]	[Ni_2_(C_14_H_11_O_2_)_4_(C_10_H_8_N_2_)_2_(H_2_O)]·2.5C_2_H_3_N·C_14_H_12_O_2_
*M*_r_	1296.74	1607.58
Crystal system, space group	Triclinic, *P* 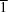	Triclinic, *P* 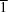
Temperature (K)	100	100
*a*, *b*, *c* (Å)	13.2138 (2), 14.1981 (2), 17.5370 (2)	15.6064 (3), 17.7440 (4), 17.9143 (4)
α, β, γ (°)	82.696 (1), 80.002 (1), 80.784 (1)	70.462 (2), 69.631 (2), 64.726 (2)
*V* (Å^3^)	3181.60 (8)	4103.17 (18)
*Z*	2	2
Radiation type	Cu *K*α	Cu *K*α
μ (mm^−1^)	1.25	1.10
Crystal size (mm)	0.21 × 0.13 × 0.07	0.22 × 0.14 × 0.08

Data collection		
Diffractometer	Rigaku SuperNova, Single source at offset/far, HyPix3000	Rigaku SuperNova, Single source at offset/far, HyPix3000
Absorption correction	Multi-scan (*CrysAlis PRO*; Rigaku OD, 2017 ▸)	Multi-scan (*CrysAlis PRO*; Rigaku OD, 2017 ▸)
*T*_min_, *T*_max_	0.815, 1.000	0.809, 1.000
No. of measured, independent and observed [*I* > 2σ(*I*)] reflections	35511, 11971, 11154	52698, 15557, 13874
*R*_int_	0.031	0.045
(sin θ/λ)_max_ (Å^−1^)	0.609	0.609

Refinement		
*R*[*F* ^2^ > 2σ(*F* ^2^)], *wR*(*F* ^2^), *S*	0.034, 0.088, 1.03	0.038, 0.103, 1.06
No. of reflections	11971	15557
No. of parameters	949	1079
No. of restraints	42	1
H-atom treatment	H atoms treated by a mixture of independent and constrained refinement	H atoms treated by a mixture of independent and constrained refinement
Δρ_max_, Δρ_min_ (e Å^−3^)	0.32, −0.44	0.48, −0.57

Computer programs: *CrysAlis PRO* (Rigaku OD, 2017 ▸), *SHELXT* (Sheldrick, 2015*a* ▸), *SHELXL* (Sheldrick, 2015*b* ▸), *DIAMOND* (Brandenburg, 2012 ▸) and *OLEX2* (Dolomanov *et al.*, 2009 ▸).

## supplementary crystallographic information

### µ-Aqua-κ2O:O-di-µ-diphenylacetato-κ4O:O'-bis[(diphenylacetato-κO)bis(pyridine-κN)nickel(II)] (1) Crystal data

**Table:**

[Ni_2_(C_14_H_11_O_2_)_4_(C_5_H_5_N)_4_(H_2_O)]	*Z* = 2
*M**_r_* = 1296.74	*F*(000) = 1356
Triclinic, *P*1	*D*_x_ = 1.354 Mg m^−^^3^
*a* = 13.2138 (2) Å	Cu *K*α radiation, λ = 1.54184 Å
*b* = 14.1981 (2) Å	Cell parameters from 25598 reflections
*c* = 17.5370 (2) Å	θ = 2.5–72.0°
α = 82.696 (1)°	µ = 1.25 mm^−^^1^
β = 80.002 (1)°	*T* = 100 K
γ = 80.784 (1)°	Prism, light green
*V* = 3181.60 (8) Å^3^	0.21 × 0.13 × 0.07 mm

### µ-Aqua-κ2O:O-di-µ-diphenylacetato-κ4O:O'-bis[(diphenylacetato-κO)bis(pyridine-κN)nickel(II)] (1) Data collection

**Table:**

SuperNova, Single source at offset/far, HyPix3000 diffractometer	11971 independent reflections
Radiation source: micro-focus sealed X-ray tube, SuperNova (Cu) X-ray Source	11154 reflections with *I* > 2σ(*I*)
Mirror monochromator	*R*_int_ = 0.031
ω scans	θ_max_ = 70.0°, θ_min_ = 2.6°
Absorption correction: multi-scan (CrysAlis PRO; Rigaku OD, 2017)	*h* = −15→16
*T*_min_ = 0.815, *T*_max_ = 1.000	*k* = −17→17
35511 measured reflections	*l* = −20→21

### µ-Aqua-κ2O:O-di-µ-diphenylacetato-κ4O:O'-bis[(diphenylacetato-κO)bis(pyridine-κN)nickel(II)] (1) Refinement

**Table:**

Refinement on *F*^2^	Secondary atom site location: difference Fourier map
Least-squares matrix: full	Hydrogen site location: mixed
*R*[*F*^2^ > 2σ(*F*^2^)] = 0.034	H atoms treated by a mixture of independent and constrained refinement
*wR*(*F*^2^) = 0.088	*w* = 1/[σ^2^(*F*_o_^2^) + (0.0414*P*)^2^ + 1.5925*P*] where *P* = (*F*_o_^2^ + 2*F*_c_^2^)/3
*S* = 1.03	(Δ/σ)_max_ < 0.001
11971 reflections	Δρ_max_ = 0.32 e Å^−^^3^
949 parameters	Δρ_min_ = −0.44 e Å^−^^3^
42 restraints	Extinction correction: (SHELXL-2018/3; Sheldrick 2015b), Fc^*^=kFc[1+0.001xFc^2^λ^3^/sin(2θ)]^-1/4^
Primary atom site location: dual	Extinction coefficient: 0.00100 (6)

### µ-Aqua-κ2O:O-di-µ-diphenylacetato-κ4O:O'-bis[(diphenylacetato-κO)bis(pyridine-κN)nickel(II)] (1) Special details

**Table:**

Geometry. All esds (except the esd in the dihedral angle between two l.s. planes) are estimated using the full covariance matrix. The cell esds are taken into account individually in the estimation of esds in distances, angles and torsion angles; correlations between esds in cell parameters are only used when they are defined by crystal symmetry. An approximate (isotropic) treatment of cell esds is used for estimating esds involving l.s. planes.

### µ-Aqua-κ2O:O-di-µ-diphenylacetato-κ4O:O'-bis[(diphenylacetato-κO)bis(pyridine-κN)nickel(II)] (1) Fractional atomic coordinates and isotropic or equivalent isotropic displacement parameters (Å^2^)

**Table:**

	*x*	*y*	*z*	*U*_iso_*/*U*_eq_	Occ. (<1)
Ni1	0.86036 (2)	0.56693 (2)	0.22819 (2)	0.01538 (7)	
Ni2	0.63704 (2)	0.71634 (2)	0.31544 (2)	0.01813 (7)	
O1	0.83261 (8)	0.42908 (7)	0.22669 (6)	0.0203 (2)	
O2	0.70924 (8)	0.41090 (8)	0.33058 (6)	0.0261 (2)	
O3	0.75989 (7)	0.61255 (7)	0.14928 (6)	0.0195 (2)	
O4	0.61190 (8)	0.69087 (8)	0.21025 (6)	0.0237 (2)	
O5	0.90084 (8)	0.69794 (7)	0.22860 (6)	0.0207 (2)	
O6	0.75619 (8)	0.79440 (8)	0.27245 (6)	0.0251 (2)	
O7	0.65566 (8)	0.75227 (7)	0.42278 (6)	0.0191 (2)	
O8	0.79419 (8)	0.64488 (7)	0.44359 (6)	0.0229 (2)	
O9	0.74232 (8)	0.58900 (8)	0.32440 (6)	0.0177 (2)	
H9A	0.7221 (16)	0.5342 (16)	0.3318 (12)	0.038 (6)*	
H9B	0.7648 (18)	0.6012 (17)	0.3656 (15)	0.051 (6)*	
N1	0.98037 (9)	0.54472 (8)	0.13464 (7)	0.0173 (2)	
N2	0.97001 (9)	0.51567 (9)	0.30433 (7)	0.0204 (3)	
N3	0.51229 (9)	0.64273 (10)	0.36897 (7)	0.0225 (3)	
N4	0.53887 (10)	0.84630 (10)	0.30576 (7)	0.0261 (3)	
C1	1.00564 (12)	0.61720 (11)	0.08151 (8)	0.0211 (3)	
H1	0.968900	0.678170	0.087035	0.025*	
C2	1.08438 (12)	0.60480 (12)	0.01866 (9)	0.0249 (3)	
H2	1.100783	0.656858	−0.016639	0.030*	
C3	1.13811 (12)	0.51440 (12)	0.00909 (9)	0.0246 (3)	
H3	1.189590	0.503962	−0.033628	0.030*	
C4	1.11372 (12)	0.43957 (11)	0.06445 (9)	0.0246 (3)	
H4	1.149642	0.378069	0.060163	0.029*	
C5	1.03497 (11)	0.45756 (10)	0.12640 (9)	0.0203 (3)	
H5	1.019396	0.407139	0.163737	0.024*	
C6	0.95013 (12)	0.45687 (11)	0.36959 (9)	0.0234 (3)	
H6	0.886801	0.433441	0.380625	0.028*	
C7	1.02039 (14)	0.42979 (12)	0.42112 (9)	0.0293 (4)	
H7	1.003993	0.389270	0.466079	0.035*	
C8	1.11481 (14)	0.46336 (12)	0.40527 (10)	0.0318 (4)	
H8	1.162727	0.446363	0.439460	0.038*	
C9	1.13695 (13)	0.52269 (13)	0.33768 (10)	0.0301 (4)	
H9	1.200396	0.545815	0.325084	0.036*	
C10	1.06261 (12)	0.54709 (11)	0.28896 (9)	0.0243 (3)	
H10	1.077663	0.587141	0.243477	0.029*	
C11	0.46124 (13)	0.66469 (14)	0.43870 (10)	0.0338 (4)	
H11	0.476910	0.717169	0.458865	0.041*	
C12	0.38673 (14)	0.61388 (16)	0.48233 (11)	0.0415 (5)	
H12	0.354059	0.631047	0.530978	0.050*	
C13	0.36170 (14)	0.53689 (15)	0.45212 (11)	0.0389 (4)	
H13	0.312726	0.500377	0.480476	0.047*	
C14	0.41074 (13)	0.51523 (13)	0.37911 (12)	0.0360 (4)	
H14	0.393621	0.465172	0.356650	0.043*	
C15	0.48575 (12)	0.56905 (12)	0.33978 (10)	0.0286 (3)	
H15	0.519249	0.553400	0.290908	0.034*	
C16	0.56963 (14)	0.92724 (12)	0.32038 (10)	0.0312 (4)	
H16	0.633690	0.923154	0.336632	0.037*	
C17	0.50984 (17)	1.01630 (14)	0.31223 (12)	0.0443 (5)	
H17	0.533711	1.070945	0.322276	0.053*	
C18	0.41458 (19)	1.02259 (16)	0.28903 (12)	0.0544 (6)	
H18	0.372650	1.081506	0.283804	0.065*	
C19	0.38216 (18)	0.94072 (18)	0.27368 (12)	0.0568 (7)	
H19	0.318144	0.943528	0.257665	0.068*	
C20	0.44654 (15)	0.85328 (15)	0.28244 (10)	0.0406 (5)	
H20	0.424539	0.798030	0.271705	0.049*	
C21	0.77488 (11)	0.38012 (10)	0.27535 (8)	0.0189 (3)	
C22	0.78915 (11)	0.27192 (10)	0.26665 (9)	0.0210 (3)	
H22	0.754956	0.241003	0.315349	0.025*	
C23	0.73781 (11)	0.24837 (11)	0.20167 (9)	0.0226 (3)	
C24	0.71461 (14)	0.15525 (13)	0.20423 (10)	0.0326 (4)	
H24	0.731182	0.110038	0.245043	0.039*	
C25	0.66754 (16)	0.12921 (15)	0.14723 (11)	0.0440 (5)	
H25	0.652025	0.067101	0.150215	0.053*	
C26	0.64343 (15)	0.19529 (16)	0.08575 (11)	0.0426 (5)	
H26	0.612092	0.177670	0.047182	0.051*	
C27	0.66614 (14)	0.28780 (15)	0.08184 (10)	0.0346 (4)	
H27	0.650024	0.332389	0.040524	0.042*	
C28	0.71314 (12)	0.31440 (12)	0.13966 (9)	0.0267 (3)	
H28	0.728062	0.376714	0.136692	0.032*	
C29	0.90422 (12)	0.23236 (10)	0.25944 (9)	0.0221 (3)	
C30	0.95005 (12)	0.21158 (11)	0.32657 (10)	0.0255 (3)	
H30	0.909515	0.219255	0.374926	0.031*	
C31	1.05549 (13)	0.17957 (12)	0.32195 (11)	0.0318 (4)	
H31	1.085284	0.165948	0.367120	0.038*	
C32	1.11661 (13)	0.16781 (12)	0.25025 (11)	0.0323 (4)	
H32	1.187411	0.146654	0.247143	0.039*	
C33	1.07188 (13)	0.18766 (12)	0.18344 (11)	0.0322 (4)	
H33	1.112617	0.179612	0.135217	0.039*	
C34	0.96625 (13)	0.21961 (12)	0.18800 (10)	0.0290 (3)	
H34	0.936723	0.232606	0.142690	0.035*	
C35	0.66876 (11)	0.65485 (10)	0.15315 (8)	0.0176 (3)	
C36	0.61982 (11)	0.66467 (11)	0.07811 (8)	0.0193 (3)	
H36	0.664399	0.618237	0.045642	0.023*	0.540 (5)
H36A	0.653560	0.613364	0.045960	0.023*	0.460 (5)
C37	0.5100 (7)	0.6270 (4)	0.0969 (4)	0.0173 (10)	0.540 (5)
C37A	0.5095 (8)	0.6602 (4)	0.0965 (6)	0.0173 (10)	0.460 (5)
C38	0.4230 (3)	0.6850 (3)	0.1284 (2)	0.0284 (8)	0.540 (5)
H38	0.427292	0.747919	0.135741	0.034*	0.540 (5)
C38A	0.4336 (3)	0.7400 (4)	0.1143 (2)	0.0291 (10)	0.460 (5)
H38A	0.453707	0.800208	0.112094	0.035*	0.460 (5)
C39	0.3275 (4)	0.6491 (5)	0.1496 (4)	0.0381 (14)	0.540 (5)
H39	0.268912	0.687149	0.172454	0.046*	0.540 (5)
C39A	0.3299 (3)	0.7294 (5)	0.1351 (2)	0.0477 (15)	0.460 (5)
H39A	0.281006	0.782242	0.147526	0.057*	0.460 (5)
C40	0.3223 (4)	0.5562 (4)	0.1356 (3)	0.0387 (11)	0.540 (5)
H40	0.259573	0.531890	0.148703	0.046*	0.540 (5)
C40A	0.2988 (6)	0.6408 (9)	0.1373 (5)	0.056 (2)	0.460 (5)
H40A	0.228615	0.634638	0.149477	0.067*	0.460 (5)
C41	0.4082 (3)	0.5006 (3)	0.1031 (2)	0.0404 (10)	0.540 (5)
H41	0.403893	0.438635	0.093266	0.048*	0.540 (5)
C41A	0.3693 (6)	0.5629 (6)	0.1219 (4)	0.057 (2)	0.460 (5)
H41A	0.348126	0.503056	0.124339	0.068*	0.460 (5)
C42	0.5024 (4)	0.5357 (3)	0.0843 (3)	0.0270 (8)	0.540 (5)
H42	0.561066	0.496604	0.062882	0.032*	0.540 (5)
C42A	0.4737 (5)	0.5730 (5)	0.1025 (3)	0.0365 (12)	0.460 (5)
H42A	0.521614	0.518524	0.093157	0.044*	0.460 (5)
C43	0.62888 (15)	0.76136 (12)	0.03166 (10)	0.0323 (4)	
C44	0.6813 (3)	0.8349 (2)	0.05213 (19)	0.0301 (5)	0.517 (2)
H44	0.705858	0.825755	0.099494	0.036*	0.517 (2)
C44A	0.5936 (3)	0.8448 (2)	0.0759 (2)	0.0301 (5)	0.483 (2)
H44A	0.574533	0.837552	0.129649	0.036*	0.483 (2)
C45	0.6961 (3)	0.9188 (3)	0.0033 (2)	0.0398 (9)	0.517 (2)
H45	0.723096	0.967273	0.020732	0.048*	0.517 (2)
C45A	0.5897 (3)	0.9347 (3)	0.0343 (2)	0.0392 (9)	0.483 (2)
H45A	0.575781	0.989377	0.060586	0.047*	0.483 (2)
C46	0.6707 (3)	0.9300 (3)	−0.0706 (2)	0.0376 (6)	0.517 (2)
H46	0.681905	0.984535	−0.104465	0.045*	0.517 (2)
C46A	0.6067 (3)	0.9435 (3)	−0.0469 (2)	0.0376 (6)	0.483 (2)
H46A	0.603167	1.004533	−0.073693	0.045*	0.483 (2)
C47	0.62988 (15)	0.85997 (15)	−0.09120 (11)	0.0418 (5)	
H47	0.646586	0.864763	−0.145162	0.050*	0.483 (2)
H47A	0.603619	0.869272	−0.137891	0.050*	0.517 (2)
C48	0.62478 (15)	0.77339 (13)	−0.04588 (10)	0.0348 (4)	
H48	0.618164	0.720343	−0.069794	0.042*	
C49	0.85089 (12)	0.77680 (11)	0.24726 (8)	0.0207 (3)	
C50	0.91418 (13)	0.86074 (11)	0.23638 (9)	0.0265 (3)	
H50A	0.863392	0.917515	0.247632	0.032*	0.493 (6)
H50	0.866623	0.921026	0.240443	0.032*	0.507 (6)
C51	0.98786 (12)	0.85182 (12)	0.29505 (10)	0.0289 (4)	
C52	1.03369 (14)	0.93196 (13)	0.30295 (14)	0.0455 (5)	
H52	1.016904	0.989673	0.273275	0.055*	
C53	1.10317 (18)	0.92623 (16)	0.3541 (2)	0.0689 (8)	
H53	1.132716	0.980115	0.358484	0.083*	
C54	1.12956 (18)	0.84128 (16)	0.39892 (18)	0.0643 (7)	
H54	1.177474	0.837490	0.432657	0.077*	
C55	1.08389 (16)	0.76237 (14)	0.39294 (13)	0.0441 (5)	
H55	1.100838	0.705022	0.423023	0.053*	
C56	1.01275 (13)	0.76792 (12)	0.34229 (10)	0.0310 (4)	
H56	0.981177	0.714559	0.339932	0.037*	
C57A	0.9471 (5)	0.8709 (4)	0.1487 (4)	0.0214 (12)	0.493 (6)
C58	0.9364 (4)	0.8885 (3)	0.0929 (3)	0.0252 (9)	0.507 (6)
H58	0.864586	0.904320	0.101713	0.030*	0.507 (6)
C58A	0.8777 (5)	0.8931 (3)	0.0952 (2)	0.0325 (11)	0.493 (6)
H58A	0.806960	0.904370	0.113644	0.039*	0.493 (6)
C59	0.9884 (4)	0.8883 (4)	0.0163 (3)	0.0275 (13)	0.507 (6)
H59	0.952210	0.902579	−0.025671	0.033*	0.507 (6)
C59A	0.9106 (6)	0.8988 (3)	0.0158 (2)	0.0488 (16)	0.493 (6)
H59A	0.863060	0.913480	−0.018737	0.059*	0.493 (6)
C60	1.0975 (5)	0.8658 (4)	0.0048 (3)	0.0285 (12)	0.507 (6)
H60	1.134467	0.865368	−0.045400	0.034*	0.507 (6)
C60A	1.0192 (8)	0.8818 (4)	−0.0118 (4)	0.053 (2)	0.493 (6)
H60A	1.043481	0.886006	−0.065037	0.064*	0.493 (6)
C61	1.1494 (3)	0.8445 (3)	0.0678 (2)	0.0336 (11)	0.507 (6)
H61	1.221338	0.830062	0.059740	0.040*	0.507 (6)
C61A	1.0857 (6)	0.8598 (4)	0.0390 (4)	0.047 (2)	0.493 (6)
H61A	1.156469	0.848324	0.020576	0.056*	0.493 (6)
C62	1.0958 (4)	0.8442 (3)	0.1427 (3)	0.0267 (9)	0.507 (6)
H62	1.131696	0.829221	0.184812	0.032*	0.507 (6)
C62A	1.0517 (5)	0.8536 (3)	0.1199 (3)	0.0321 (11)	0.493 (6)
H62A	1.099807	0.837728	0.154044	0.038*	0.493 (6)
C63	0.73115 (11)	0.71922 (10)	0.45772 (8)	0.0169 (3)	
C64	0.74739 (11)	0.77306 (10)	0.52482 (8)	0.0186 (3)	
H64	0.818837	0.751505	0.534194	0.022*	
C65	0.73824 (12)	0.88095 (11)	0.50243 (8)	0.0217 (3)	
C66	0.64628 (13)	0.94269 (12)	0.51620 (11)	0.0337 (4)	
H66	0.586291	0.918012	0.540319	0.040*	
C67	0.64156 (15)	1.04079 (13)	0.49477 (12)	0.0376 (4)	
H67	0.579133	1.081071	0.505385	0.045*	
C68	0.72881 (17)	1.07819 (13)	0.45802 (12)	0.0420 (5)	
H68	0.725632	1.143498	0.442153	0.050*	
C69	0.8211 (2)	1.01800 (16)	0.44490 (16)	0.0665 (8)	
H69	0.880727	1.043063	0.420559	0.080*	
C70	0.82635 (16)	0.92010 (14)	0.46756 (13)	0.0471 (5)	
H70	0.889706	0.880598	0.459194	0.057*	
C71	0.67644 (11)	0.74405 (10)	0.59961 (8)	0.0187 (3)	
C72	0.71774 (12)	0.71662 (11)	0.66819 (9)	0.0223 (3)	
H72	0.788675	0.714107	0.667412	0.027*	
C73	0.65462 (14)	0.69292 (12)	0.73784 (9)	0.0290 (4)	
H73	0.683411	0.675599	0.783255	0.035*	
C74	0.54887 (14)	0.69499 (12)	0.73991 (10)	0.0310 (4)	
H74	0.506420	0.679775	0.786550	0.037*	
C75	0.50733 (13)	0.71997 (12)	0.67172 (10)	0.0281 (3)	
H75	0.436755	0.720253	0.672398	0.034*	
C76	0.57012 (12)	0.74460 (11)	0.60234 (9)	0.0231 (3)	
H76	0.540993	0.761701	0.557060	0.028*	
C57	0.9883 (5)	0.8662 (4)	0.1552 (4)	0.0200 (12)	0.507 (6)

### µ-Aqua-κ2O:O-di-µ-diphenylacetato-κ4O:O'-bis[(diphenylacetato-κO)bis(pyridine-κN)nickel(II)] (1) Atomic displacement parameters (Å^2^)

**Table:**

	*U*^11^	*U*^22^	*U*^33^	*U*^12^	*U*^13^	*U*^23^
Ni1	0.01573 (13)	0.01719 (13)	0.01225 (12)	0.00063 (9)	−0.00144 (9)	−0.00258 (9)
Ni2	0.01713 (13)	0.02215 (14)	0.01389 (13)	0.00304 (10)	−0.00201 (9)	−0.00521 (10)
O1	0.0222 (5)	0.0189 (5)	0.0175 (5)	−0.0023 (4)	0.0034 (4)	−0.0022 (4)
O2	0.0244 (5)	0.0299 (6)	0.0227 (6)	−0.0069 (4)	0.0078 (4)	−0.0090 (4)
O3	0.0177 (5)	0.0245 (5)	0.0155 (5)	0.0017 (4)	−0.0033 (4)	−0.0041 (4)
O4	0.0216 (5)	0.0334 (6)	0.0146 (5)	0.0062 (4)	−0.0042 (4)	−0.0074 (4)
O5	0.0221 (5)	0.0202 (5)	0.0192 (5)	−0.0005 (4)	−0.0004 (4)	−0.0063 (4)
O6	0.0244 (6)	0.0230 (5)	0.0245 (6)	0.0018 (4)	0.0016 (4)	−0.0036 (4)
O7	0.0185 (5)	0.0234 (5)	0.0153 (5)	0.0003 (4)	−0.0027 (4)	−0.0056 (4)
O8	0.0260 (5)	0.0243 (5)	0.0175 (5)	0.0042 (4)	−0.0055 (4)	−0.0051 (4)
O9	0.0188 (5)	0.0202 (5)	0.0132 (5)	0.0005 (4)	−0.0013 (4)	−0.0040 (4)
N1	0.0173 (6)	0.0204 (6)	0.0144 (6)	−0.0019 (5)	−0.0024 (5)	−0.0033 (5)
N2	0.0208 (6)	0.0225 (6)	0.0166 (6)	0.0026 (5)	−0.0032 (5)	−0.0040 (5)
N3	0.0168 (6)	0.0318 (7)	0.0197 (6)	−0.0012 (5)	−0.0034 (5)	−0.0076 (5)
N4	0.0280 (7)	0.0299 (7)	0.0155 (6)	0.0096 (5)	−0.0013 (5)	−0.0041 (5)
C1	0.0248 (7)	0.0211 (7)	0.0164 (7)	−0.0017 (6)	−0.0020 (6)	−0.0021 (5)
C2	0.0273 (8)	0.0283 (8)	0.0183 (7)	−0.0070 (6)	0.0002 (6)	−0.0006 (6)
C3	0.0187 (7)	0.0351 (9)	0.0195 (8)	−0.0049 (6)	0.0031 (6)	−0.0078 (6)
C4	0.0185 (7)	0.0246 (8)	0.0289 (8)	−0.0001 (6)	0.0020 (6)	−0.0079 (6)
C5	0.0190 (7)	0.0200 (7)	0.0209 (7)	−0.0023 (6)	−0.0007 (6)	−0.0025 (6)
C6	0.0266 (8)	0.0233 (7)	0.0178 (7)	0.0022 (6)	−0.0017 (6)	−0.0023 (6)
C7	0.0404 (9)	0.0252 (8)	0.0190 (8)	0.0071 (7)	−0.0073 (7)	−0.0010 (6)
C8	0.0343 (9)	0.0336 (9)	0.0283 (9)	0.0098 (7)	−0.0175 (7)	−0.0076 (7)
C9	0.0240 (8)	0.0359 (9)	0.0314 (9)	0.0015 (7)	−0.0094 (7)	−0.0073 (7)
C10	0.0221 (7)	0.0283 (8)	0.0216 (8)	0.0006 (6)	−0.0041 (6)	−0.0037 (6)
C11	0.0251 (8)	0.0566 (11)	0.0237 (8)	−0.0136 (8)	0.0012 (7)	−0.0163 (8)
C12	0.0284 (9)	0.0741 (14)	0.0252 (9)	−0.0189 (9)	0.0016 (7)	−0.0102 (9)
C13	0.0229 (8)	0.0519 (11)	0.0422 (11)	−0.0140 (8)	−0.0042 (8)	0.0036 (9)
C14	0.0234 (8)	0.0344 (9)	0.0521 (12)	−0.0053 (7)	−0.0045 (8)	−0.0122 (8)
C15	0.0217 (8)	0.0330 (9)	0.0318 (9)	−0.0008 (6)	−0.0021 (6)	−0.0131 (7)
C16	0.0348 (9)	0.0230 (8)	0.0273 (9)	0.0041 (7)	0.0091 (7)	0.0004 (6)
C17	0.0524 (12)	0.0270 (9)	0.0398 (11)	0.0091 (8)	0.0109 (9)	0.0045 (8)
C18	0.0714 (15)	0.0427 (12)	0.0328 (11)	0.0346 (11)	−0.0045 (10)	−0.0012 (9)
C19	0.0539 (13)	0.0746 (16)	0.0350 (11)	0.0389 (12)	−0.0232 (10)	−0.0181 (10)
C20	0.0398 (10)	0.0512 (12)	0.0291 (9)	0.0206 (9)	−0.0154 (8)	−0.0176 (8)
C21	0.0165 (7)	0.0234 (7)	0.0167 (7)	−0.0023 (6)	−0.0016 (5)	−0.0032 (6)
C22	0.0205 (7)	0.0216 (7)	0.0187 (7)	−0.0062 (6)	0.0041 (6)	−0.0001 (6)
C23	0.0178 (7)	0.0279 (8)	0.0208 (8)	−0.0072 (6)	0.0077 (6)	−0.0069 (6)
C24	0.0365 (9)	0.0314 (9)	0.0289 (9)	−0.0130 (7)	0.0083 (7)	−0.0081 (7)
C25	0.0534 (12)	0.0477 (11)	0.0348 (10)	−0.0288 (10)	0.0141 (9)	−0.0205 (9)
C26	0.0399 (10)	0.0666 (13)	0.0272 (9)	−0.0264 (10)	0.0087 (8)	−0.0231 (9)
C27	0.0301 (9)	0.0550 (11)	0.0188 (8)	−0.0113 (8)	0.0035 (7)	−0.0079 (7)
C28	0.0250 (8)	0.0333 (9)	0.0214 (8)	−0.0086 (7)	0.0039 (6)	−0.0060 (6)
C29	0.0219 (7)	0.0145 (7)	0.0287 (8)	−0.0052 (6)	0.0011 (6)	−0.0006 (6)
C30	0.0278 (8)	0.0210 (7)	0.0291 (8)	−0.0099 (6)	−0.0016 (6)	−0.0034 (6)
C31	0.0319 (9)	0.0257 (8)	0.0425 (10)	−0.0084 (7)	−0.0133 (8)	−0.0053 (7)
C32	0.0215 (8)	0.0256 (8)	0.0508 (11)	−0.0039 (6)	−0.0052 (7)	−0.0074 (7)
C33	0.0255 (8)	0.0297 (9)	0.0366 (10)	−0.0007 (7)	0.0050 (7)	−0.0033 (7)
C34	0.0253 (8)	0.0303 (8)	0.0268 (8)	−0.0002 (7)	0.0017 (6)	0.0013 (7)
C35	0.0196 (7)	0.0188 (7)	0.0141 (7)	−0.0016 (5)	−0.0027 (5)	−0.0020 (5)
C36	0.0187 (7)	0.0247 (7)	0.0141 (7)	0.0011 (6)	−0.0032 (5)	−0.0055 (6)
C37	0.0245 (9)	0.016 (3)	0.0140 (8)	−0.012 (3)	−0.0079 (6)	0.005 (3)
C37A	0.0245 (9)	0.016 (3)	0.0140 (8)	−0.012 (3)	−0.0079 (6)	0.005 (3)
C38	0.0255 (17)	0.033 (2)	0.0292 (18)	−0.0031 (15)	−0.0049 (13)	−0.0114 (16)
C38A	0.0189 (18)	0.044 (3)	0.026 (2)	−0.0020 (18)	−0.0044 (14)	−0.0091 (19)
C39	0.030 (3)	0.059 (3)	0.029 (3)	−0.010 (2)	−0.006 (2)	−0.009 (2)
C39A	0.019 (2)	0.097 (4)	0.026 (2)	−0.010 (2)	−0.0017 (15)	−0.002 (2)
C40	0.034 (2)	0.058 (3)	0.027 (2)	−0.024 (2)	−0.0055 (19)	0.0066 (17)
C40A	0.035 (4)	0.114 (6)	0.025 (3)	−0.039 (3)	−0.011 (2)	0.014 (3)
C41	0.051 (2)	0.036 (2)	0.039 (2)	−0.0198 (18)	−0.0113 (17)	0.0018 (16)
C41A	0.049 (4)	0.085 (5)	0.043 (3)	−0.047 (4)	−0.015 (3)	0.027 (3)
C42	0.033 (2)	0.022 (2)	0.028 (2)	−0.0053 (16)	−0.0092 (15)	−0.0006 (15)
C42A	0.043 (3)	0.033 (3)	0.037 (3)	−0.012 (3)	−0.016 (2)	0.007 (2)
C43	0.0473 (10)	0.0255 (8)	0.0275 (9)	−0.0023 (7)	−0.0195 (8)	−0.0001 (7)
C44	0.0386 (12)	0.0282 (12)	0.0260 (12)	−0.0098 (11)	−0.0116 (10)	0.0033 (9)
C44A	0.0386 (12)	0.0282 (12)	0.0260 (12)	−0.0098 (11)	−0.0116 (10)	0.0033 (9)
C45	0.052 (2)	0.0315 (18)	0.038 (2)	−0.0154 (16)	−0.0127 (17)	0.0079 (15)
C45A	0.047 (2)	0.0240 (18)	0.050 (2)	−0.0102 (16)	−0.0157 (19)	0.0026 (16)
C46	0.0359 (16)	0.0355 (14)	0.0366 (16)	−0.0059 (14)	−0.0051 (13)	0.0149 (12)
C46A	0.0359 (16)	0.0355 (14)	0.0366 (16)	−0.0059 (14)	−0.0051 (13)	0.0149 (12)
C47	0.0412 (10)	0.0473 (11)	0.0311 (10)	−0.0032 (9)	−0.0050 (8)	0.0129 (8)
C48	0.0439 (10)	0.0362 (9)	0.0213 (8)	−0.0044 (8)	0.0008 (7)	−0.0010 (7)
C49	0.0266 (8)	0.0216 (7)	0.0124 (7)	0.0001 (6)	−0.0015 (6)	−0.0030 (5)
C50	0.0348 (9)	0.0184 (7)	0.0222 (8)	−0.0023 (6)	0.0067 (7)	−0.0040 (6)
C51	0.0213 (8)	0.0241 (8)	0.0396 (10)	−0.0062 (6)	0.0055 (7)	−0.0070 (7)
C52	0.0275 (9)	0.0251 (9)	0.0838 (16)	−0.0099 (7)	−0.0053 (9)	−0.0018 (9)
C53	0.0428 (12)	0.0311 (11)	0.145 (3)	−0.0148 (9)	−0.0387 (15)	−0.0076 (13)
C54	0.0492 (13)	0.0400 (12)	0.118 (2)	−0.0116 (10)	−0.0452 (14)	−0.0104 (13)
C55	0.0428 (11)	0.0332 (10)	0.0619 (13)	−0.0087 (8)	−0.0200 (10)	−0.0054 (9)
C56	0.0306 (9)	0.0268 (8)	0.0374 (10)	−0.0106 (7)	−0.0025 (7)	−0.0062 (7)
C57A	0.019 (3)	0.0159 (17)	0.026 (3)	−0.006 (2)	0.009 (3)	−0.0029 (15)
C58	0.021 (2)	0.0274 (18)	0.026 (3)	−0.0064 (16)	0.0028 (19)	−0.0003 (14)
C58A	0.043 (3)	0.0280 (19)	0.028 (2)	−0.0087 (19)	−0.007 (2)	−0.0021 (14)
C59	0.029 (4)	0.027 (2)	0.024 (3)	−0.0072 (18)	0.003 (2)	0.000 (2)
C59A	0.089 (5)	0.032 (2)	0.031 (2)	−0.019 (2)	−0.017 (2)	0.0012 (16)
C60	0.032 (3)	0.0290 (19)	0.021 (3)	−0.0107 (17)	0.013 (2)	−0.005 (2)
C60A	0.094 (7)	0.030 (2)	0.030 (4)	−0.025 (3)	0.024 (4)	−0.008 (2)
C61	0.026 (2)	0.042 (2)	0.032 (2)	−0.0077 (15)	0.0047 (17)	−0.0078 (15)
C61A	0.059 (5)	0.038 (3)	0.036 (4)	−0.022 (2)	0.035 (4)	−0.014 (3)
C62	0.017 (2)	0.036 (2)	0.025 (2)	−0.0029 (16)	0.0049 (16)	−0.0056 (14)
C62A	0.023 (3)	0.0269 (19)	0.043 (3)	−0.0038 (17)	0.005 (3)	−0.0043 (17)
C63	0.0176 (7)	0.0209 (7)	0.0115 (6)	−0.0058 (5)	0.0021 (5)	−0.0009 (5)
C64	0.0168 (7)	0.0253 (7)	0.0144 (7)	−0.0061 (6)	−0.0004 (5)	−0.0037 (5)
C65	0.0271 (8)	0.0257 (8)	0.0141 (7)	−0.0107 (6)	−0.0005 (6)	−0.0036 (6)
C66	0.0263 (8)	0.0284 (9)	0.0461 (11)	−0.0093 (7)	−0.0049 (7)	0.0026 (7)
C67	0.0372 (10)	0.0284 (9)	0.0479 (11)	−0.0052 (7)	−0.0110 (8)	0.0008 (8)
C68	0.0603 (13)	0.0251 (9)	0.0394 (11)	−0.0160 (9)	0.0023 (9)	−0.0003 (8)
C69	0.0613 (15)	0.0348 (11)	0.0884 (19)	−0.0221 (11)	0.0407 (13)	−0.0024 (11)
C70	0.0402 (11)	0.0307 (10)	0.0612 (14)	−0.0127 (8)	0.0254 (10)	−0.0058 (9)
C71	0.0227 (7)	0.0190 (7)	0.0152 (7)	−0.0071 (6)	0.0013 (6)	−0.0059 (5)
C72	0.0254 (8)	0.0251 (8)	0.0180 (7)	−0.0085 (6)	−0.0012 (6)	−0.0048 (6)
C73	0.0399 (9)	0.0322 (9)	0.0157 (7)	−0.0110 (7)	−0.0009 (7)	−0.0021 (6)
C74	0.0364 (9)	0.0333 (9)	0.0211 (8)	−0.0130 (7)	0.0110 (7)	−0.0061 (7)
C75	0.0232 (8)	0.0330 (9)	0.0279 (9)	−0.0099 (7)	0.0070 (6)	−0.0097 (7)
C76	0.0227 (7)	0.0274 (8)	0.0202 (8)	−0.0056 (6)	0.0001 (6)	−0.0082 (6)
C57	0.012 (3)	0.0188 (17)	0.028 (2)	−0.007 (2)	0.008 (3)	−0.0050 (14)

### µ-Aqua-κ2O:O-di-µ-diphenylacetato-κ4O:O'-bis[(diphenylacetato-κO)bis(pyridine-κN)nickel(II)] (1) Geometric parameters (Å, º)

**Table:**

Ni1—O1	2.0524 (10)	C38—H38	0.9300
Ni1—O3	2.0570 (10)	C38—C39	1.409 (7)
Ni1—O5	2.0174 (10)	C38A—H38A	0.9300
Ni1—O9	2.1077 (10)	C38A—C39A	1.382 (6)
Ni1—N1	2.0913 (12)	C39—H39	0.9300
Ni1—N2	2.1211 (12)	C39—C40	1.386 (9)
Ni2—O4	2.0163 (10)	C39A—H39A	0.9300
Ni2—O6	2.0518 (11)	C39A—C40A	1.379 (12)
Ni2—O7	2.0759 (10)	C40—H40	0.9300
Ni2—O9	2.1040 (10)	C40—C41	1.357 (7)
Ni2—N3	2.1109 (13)	C40A—H40A	0.9300
Ni2—N4	2.0849 (13)	C40A—C41A	1.350 (13)
O1—C21	1.2595 (17)	C41—H41	0.9300
O2—C21	1.2553 (18)	C41—C42	1.385 (6)
O3—C35	1.2512 (17)	C41A—H41A	0.9300
O4—C35	1.2548 (17)	C41A—C42A	1.388 (9)
O5—C49	1.2561 (18)	C42—H42	0.9300
O6—C49	1.2488 (18)	C42A—H42A	0.9300
O7—C63	1.2569 (17)	C43—C44	1.454 (4)
O8—C63	1.2579 (17)	C43—C44A	1.467 (4)
O9—H9A	0.85 (2)	C43—C48	1.358 (2)
O9—H9B	0.87 (3)	C44—H44	0.9300
N1—C1	1.3405 (19)	C44—C45	1.395 (4)
N1—C5	1.3401 (19)	C44A—H44A	0.9300
N2—C6	1.3412 (19)	C44A—C45A	1.386 (5)
N2—C10	1.341 (2)	C45—H45	0.9300
N3—C11	1.338 (2)	C45—C46	1.378 (5)
N3—C15	1.340 (2)	C45A—H45A	0.9300
N4—C16	1.346 (2)	C45A—C46A	1.394 (6)
N4—C20	1.338 (2)	C46—H46	0.9300
C1—H1	0.9300	C46—C47	1.319 (4)
C1—C2	1.386 (2)	C46A—H46A	0.9300
C2—H2	0.9300	C46A—C47	1.463 (5)
C2—C3	1.379 (2)	C47—H47	0.9300
C3—H3	0.9300	C47—H47A	0.9300
C3—C4	1.384 (2)	C47—C48	1.381 (3)
C4—H4	0.9300	C48—H48	0.9300
C4—C5	1.386 (2)	C49—C50	1.537 (2)
C5—H5	0.9300	C50—H50A	0.9800
C6—H6	0.9300	C50—H50	0.9800
C6—C7	1.384 (2)	C50—C51	1.516 (2)
C7—H7	0.9300	C50—C57A	1.518 (7)
C7—C8	1.377 (3)	C50—C57	1.583 (6)
C8—H8	0.9300	C51—C52	1.404 (2)
C8—C9	1.380 (3)	C51—C56	1.390 (2)
C9—H9	0.9300	C52—H52	0.9300
C9—C10	1.386 (2)	C52—C53	1.377 (3)
C10—H10	0.9300	C53—H53	0.9300
C11—H11	0.9300	C53—C54	1.384 (3)
C11—C12	1.379 (3)	C54—H54	0.9300
C12—H12	0.9300	C54—C55	1.378 (3)
C12—C13	1.382 (3)	C55—H55	0.9300
C13—H13	0.9300	C55—C56	1.388 (3)
C13—C14	1.379 (3)	C56—H56	0.9300
C14—H14	0.9300	C57A—C58A	1.397 (7)
C14—C15	1.383 (2)	C57A—C62A	1.381 (6)
C15—H15	0.9300	C58—H58	0.9300
C16—H16	0.9300	C58—C59	1.399 (7)
C16—C17	1.384 (2)	C58—C57	1.366 (7)
C17—H17	0.9300	C58A—H58A	0.9300
C17—C18	1.375 (3)	C58A—C59A	1.380 (6)
C18—H18	0.9300	C59—H59	0.9300
C18—C19	1.373 (4)	C59—C60	1.411 (8)
C19—H19	0.9300	C59A—H59A	0.9300
C19—C20	1.395 (3)	C59A—C60A	1.424 (9)
C20—H20	0.9300	C60—H60	0.9300
C21—C22	1.541 (2)	C60—C61	1.375 (7)
C22—H22	0.9800	C60A—H60A	0.9300
C22—C23	1.519 (2)	C60A—C61A	1.335 (12)
C22—C29	1.524 (2)	C61—H61	0.9300
C23—C24	1.398 (2)	C61—C62	1.380 (5)
C23—C28	1.391 (2)	C61A—H61A	0.9300
C24—H24	0.9300	C61A—C62A	1.407 (8)
C24—C25	1.381 (3)	C62—H62	0.9300
C25—H25	0.9300	C62—C57	1.389 (6)
C25—C26	1.382 (3)	C62A—H62A	0.9300
C26—H26	0.9300	C63—C64	1.5441 (19)
C26—C27	1.384 (3)	C64—H64	0.9800
C27—H27	0.9300	C64—C65	1.523 (2)
C27—C28	1.396 (2)	C64—C71	1.5259 (19)
C28—H28	0.9300	C65—C66	1.384 (2)
C29—C30	1.393 (2)	C65—C70	1.383 (2)
C29—C34	1.388 (2)	C66—H66	0.9300
C30—H30	0.9300	C66—C67	1.390 (2)
C30—C31	1.386 (2)	C67—H67	0.9300
C31—H31	0.9300	C67—C68	1.370 (3)
C31—C32	1.385 (3)	C68—H68	0.9300
C32—H32	0.9300	C68—C69	1.374 (3)
C32—C33	1.380 (3)	C69—H69	0.9300
C33—H33	0.9300	C69—C70	1.390 (3)
C33—C34	1.389 (2)	C70—H70	0.9300
C34—H34	0.9300	C71—C72	1.392 (2)
C35—C36	1.5457 (18)	C71—C76	1.396 (2)
C36—H36	0.9800	C72—H72	0.9300
C36—H36A	0.9800	C72—C73	1.390 (2)
C36—C37	1.593 (7)	C73—H73	0.9300
C36—C37A	1.447 (11)	C73—C74	1.387 (2)
C36—C43	1.516 (2)	C74—H74	0.9300
C37—C38	1.377 (9)	C74—C75	1.384 (2)
C37—C42	1.365 (5)	C75—H75	0.9300
C37A—C38A	1.415 (9)	C75—C76	1.387 (2)
C37A—C42A	1.382 (6)	C76—H76	0.9300
			
O1—Ni1—O3	89.05 (4)	C42A—C37A—C36	119.7 (6)
O1—Ni1—O9	91.80 (4)	C42A—C37A—C38A	116.1 (8)
O1—Ni1—N1	88.57 (4)	C37—C38—H38	120.0
O1—Ni1—N2	89.66 (5)	C37—C38—C39	120.0 (4)
O3—Ni1—O9	92.91 (4)	C39—C38—H38	120.0
O3—Ni1—N1	88.54 (4)	C37A—C38A—H38A	119.6
O3—Ni1—N2	176.62 (4)	C39A—C38A—C37A	120.9 (6)
O5—Ni1—O1	175.04 (4)	C39A—C38A—H38A	119.6
O5—Ni1—O3	94.32 (4)	C38—C39—H39	120.6
O5—Ni1—O9	91.66 (4)	C40—C39—C38	118.9 (5)
O5—Ni1—N1	87.87 (4)	C40—C39—H39	120.6
O5—Ni1—N2	86.77 (5)	C38A—C39A—H39A	119.9
O9—Ni1—N2	90.26 (4)	C40A—C39A—C38A	120.2 (6)
N1—Ni1—O9	178.51 (4)	C40A—C39A—H39A	119.9
N1—Ni1—N2	88.30 (5)	C39—C40—H40	119.8
O4—Ni2—O6	95.12 (5)	C41—C40—C39	120.4 (5)
O4—Ni2—O7	175.70 (4)	C41—C40—H40	119.8
O4—Ni2—O9	91.62 (4)	C39A—C40A—H40A	119.7
O4—Ni2—N3	89.72 (5)	C41A—C40A—C39A	120.5 (7)
O4—Ni2—N4	89.26 (5)	C41A—C40A—H40A	119.7
O6—Ni2—O7	84.55 (4)	C40—C41—H41	119.9
O6—Ni2—O9	91.13 (4)	C40—C41—C42	120.3 (4)
O6—Ni2—N3	174.71 (5)	C42—C41—H41	119.9
O6—Ni2—N4	86.13 (5)	C40A—C41A—H41A	120.3
O7—Ni2—O9	92.67 (4)	C40A—C41A—C42A	119.4 (7)
O7—Ni2—N3	90.47 (4)	C42A—C41A—H41A	120.3
O7—Ni2—N4	86.44 (5)	C37—C42—C41	120.8 (5)
O9—Ni2—N3	90.86 (5)	C37—C42—H42	119.6
N4—Ni2—O9	177.19 (5)	C41—C42—H42	119.6
N4—Ni2—N3	91.81 (5)	C37A—C42A—C41A	122.9 (8)
C21—O1—Ni1	129.29 (9)	C37A—C42A—H42A	118.5
C35—O3—Ni1	134.83 (9)	C41A—C42A—H42A	118.5
C35—O4—Ni2	133.74 (9)	C44—C43—C36	124.90 (17)
C49—O5—Ni1	133.34 (10)	C44A—C43—C36	115.38 (19)
C49—O6—Ni2	136.62 (10)	C48—C43—C36	120.72 (15)
C63—O7—Ni2	125.14 (9)	C48—C43—C44	110.06 (19)
Ni1—O9—H9A	99.6 (14)	C48—C43—C44A	117.98 (19)
Ni1—O9—H9B	113.6 (15)	C43—C44—H44	118.8
Ni2—O9—Ni1	116.32 (5)	C45—C44—C43	122.4 (3)
Ni2—O9—H9A	121.7 (14)	C45—C44—H44	118.8
Ni2—O9—H9B	95.8 (15)	C43—C44A—H44A	121.3
H9A—O9—H9B	110 (2)	C45A—C44A—C43	117.4 (3)
C1—N1—Ni1	121.13 (10)	C45A—C44A—H44A	121.3
C5—N1—Ni1	120.90 (10)	C44—C45—H45	119.9
C5—N1—C1	117.97 (13)	C46—C45—C44	120.2 (3)
C6—N2—Ni1	123.53 (10)	C46—C45—H45	119.9
C10—N2—Ni1	118.69 (10)	C44A—C45A—H45A	119.9
C10—N2—C6	117.69 (13)	C44A—C45A—C46A	120.2 (4)
C11—N3—Ni2	119.23 (11)	C46A—C45A—H45A	119.9
C11—N3—C15	117.13 (14)	C45—C46—H46	121.4
C15—N3—Ni2	123.43 (11)	C47—C46—C45	117.2 (3)
C16—N4—Ni2	119.84 (11)	C47—C46—H46	121.4
C20—N4—Ni2	122.14 (13)	C45A—C46A—H46A	118.9
C20—N4—C16	117.97 (15)	C45A—C46A—C47	122.2 (3)
N1—C1—H1	118.7	C47—C46A—H46A	118.9
N1—C1—C2	122.56 (14)	C46—C47—H47A	118.9
C2—C1—H1	118.7	C46—C47—C48	122.3 (2)
C1—C2—H2	120.4	C46A—C47—H47	122.9
C3—C2—C1	119.23 (14)	C48—C47—C46A	114.1 (2)
C3—C2—H2	120.4	C48—C47—H47	122.9
C2—C3—H3	120.7	C48—C47—H47A	118.9
C2—C3—C4	118.51 (14)	C43—C48—C47	123.68 (18)
C4—C3—H3	120.7	C43—C48—H48	118.2
C3—C4—H4	120.5	C47—C48—H48	118.2
C3—C4—C5	119.05 (14)	O5—C49—C50	115.65 (13)
C5—C4—H4	120.5	O6—C49—O5	127.33 (14)
N1—C5—C4	122.63 (14)	O6—C49—C50	117.02 (13)
N1—C5—H5	118.7	C49—C50—H50A	105.5
C4—C5—H5	118.7	C49—C50—H50	109.3
N2—C6—H6	118.8	C49—C50—C57	112.3 (2)
N2—C6—C7	122.41 (15)	C51—C50—C49	113.04 (13)
C7—C6—H6	118.8	C51—C50—H50A	105.5
C6—C7—H7	120.3	C51—C50—H50	109.3
C8—C7—C6	119.45 (15)	C51—C50—C57A	124.4 (3)
C8—C7—H7	120.3	C51—C50—C57	103.5 (3)
C7—C8—H8	120.6	C57A—C50—C49	101.3 (2)
C7—C8—C9	118.74 (15)	C57A—C50—H50A	105.5
C9—C8—H8	120.6	C57—C50—H50	109.3
C8—C9—H9	120.7	C52—C51—C50	118.99 (16)
C8—C9—C10	118.60 (16)	C56—C51—C50	123.43 (14)
C10—C9—H9	120.7	C56—C51—C52	117.58 (17)
N2—C10—C9	123.09 (15)	C51—C52—H52	119.6
N2—C10—H10	118.5	C53—C52—C51	120.86 (19)
C9—C10—H10	118.5	C53—C52—H52	119.6
N3—C11—H11	118.2	C52—C53—H53	119.6
N3—C11—C12	123.68 (17)	C52—C53—C54	120.79 (18)
C12—C11—H11	118.2	C54—C53—H53	119.6
C11—C12—H12	120.8	C53—C54—H54	120.4
C11—C12—C13	118.48 (17)	C55—C54—C53	119.1 (2)
C13—C12—H12	120.8	C55—C54—H54	120.4
C12—C13—H13	120.6	C54—C55—H55	119.8
C14—C13—C12	118.72 (17)	C54—C55—C56	120.4 (2)
C14—C13—H13	120.6	C56—C55—H55	119.8
C13—C14—H14	120.5	C51—C56—H56	119.4
C13—C14—C15	119.02 (17)	C55—C56—C51	121.16 (16)
C15—C14—H14	120.5	C55—C56—H56	119.4
N3—C15—C14	122.91 (16)	C58A—C57A—C50	124.0 (4)
N3—C15—H15	118.5	C62A—C57A—C50	118.1 (5)
C14—C15—H15	118.5	C62A—C57A—C58A	117.8 (5)
N4—C16—H16	118.6	C59—C58—H58	119.1
N4—C16—C17	122.71 (19)	C57—C58—H58	119.1
C17—C16—H16	118.6	C57—C58—C59	121.8 (4)
C16—C17—H17	120.6	C57A—C58A—H58A	118.9
C18—C17—C16	118.9 (2)	C59A—C58A—C57A	122.3 (6)
C18—C17—H17	120.6	C59A—C58A—H58A	118.9
C17—C18—H18	120.4	C58—C59—H59	121.1
C19—C18—C17	119.14 (18)	C58—C59—C60	117.8 (5)
C19—C18—H18	120.4	C60—C59—H59	121.1
C18—C19—H19	120.4	C58A—C59A—H59A	120.8
C18—C19—C20	119.1 (2)	C58A—C59A—C60A	118.5 (5)
C20—C19—H19	120.4	C60A—C59A—H59A	120.8
N4—C20—C19	122.2 (2)	C59—C60—H60	120.0
N4—C20—H20	118.9	C61—C60—C59	120.1 (5)
C19—C20—H20	118.9	C61—C60—H60	120.0
O1—C21—C22	116.15 (12)	C59A—C60A—H60A	120.2
O2—C21—O1	126.15 (14)	C61A—C60A—C59A	119.6 (6)
O2—C21—C22	117.69 (13)	C61A—C60A—H60A	120.2
C21—C22—H22	106.9	C60—C61—H61	119.6
C23—C22—C21	113.29 (12)	C60—C61—C62	120.8 (4)
C23—C22—H22	106.9	C62—C61—H61	119.6
C23—C22—C29	113.40 (12)	C60A—C61A—H61A	119.1
C29—C22—C21	109.11 (11)	C60A—C61A—C62A	121.8 (7)
C29—C22—H22	106.9	C62A—C61A—H61A	119.1
C24—C23—C22	118.21 (14)	C61—C62—H62	120.0
C28—C23—C22	123.48 (14)	C61—C62—C57	120.0 (5)
C28—C23—C24	118.31 (15)	C57—C62—H62	120.0
C23—C24—H24	119.4	C57A—C62A—C61A	120.2 (6)
C25—C24—C23	121.17 (18)	C57A—C62A—H62A	119.9
C25—C24—H24	119.4	C61A—C62A—H62A	119.9
C24—C25—H25	119.9	O7—C63—O8	125.65 (13)
C24—C25—C26	120.12 (17)	O7—C63—C64	117.57 (12)
C26—C25—H25	119.9	O8—C63—C64	116.77 (12)
C25—C26—H26	120.2	C63—C64—H64	106.8
C25—C26—C27	119.70 (17)	C65—C64—C63	111.78 (11)
C27—C26—H26	120.2	C65—C64—H64	106.8
C26—C27—H27	119.9	C65—C64—C71	113.92 (12)
C26—C27—C28	120.25 (18)	C71—C64—C63	110.41 (11)
C28—C27—H27	119.9	C71—C64—H64	106.8
C23—C28—C27	120.45 (16)	C66—C65—C64	123.43 (13)
C23—C28—H28	119.8	C70—C65—C64	118.73 (15)
C27—C28—H28	119.8	C70—C65—C66	117.83 (16)
C30—C29—C22	119.00 (14)	C65—C66—H66	119.3
C34—C29—C22	122.36 (14)	C65—C66—C67	121.46 (16)
C34—C29—C30	118.59 (15)	C67—C66—H66	119.3
C29—C30—H30	119.7	C66—C67—H67	120.0
C31—C30—C29	120.61 (16)	C68—C67—C66	120.06 (18)
C31—C30—H30	119.7	C68—C67—H67	120.0
C30—C31—H31	119.9	C67—C68—H68	120.4
C32—C31—C30	120.20 (16)	C67—C68—C69	119.17 (17)
C32—C31—H31	119.9	C69—C68—H68	120.4
C31—C32—H32	120.2	C68—C69—H69	119.6
C33—C32—C31	119.65 (16)	C68—C69—C70	120.85 (19)
C33—C32—H32	120.2	C70—C69—H69	119.6
C32—C33—H33	119.9	C65—C70—C69	120.59 (19)
C32—C33—C34	120.22 (16)	C65—C70—H70	119.7
C34—C33—H33	119.9	C69—C70—H70	119.7
C29—C34—C33	120.73 (16)	C72—C71—C64	119.61 (13)
C29—C34—H34	119.6	C72—C71—C76	118.06 (13)
C33—C34—H34	119.6	C76—C71—C64	122.33 (13)
O3—C35—O4	128.23 (13)	C71—C72—H72	119.5
O3—C35—C36	116.15 (12)	C73—C72—C71	121.02 (15)
O4—C35—C36	115.62 (12)	C73—C72—H72	119.5
C35—C36—H36	104.8	C72—C73—H73	119.9
C35—C36—H36A	109.7	C74—C73—C72	120.29 (15)
C35—C36—C37	110.0 (3)	C74—C73—H73	119.9
C37—C36—H36	104.8	C73—C74—H74	120.4
C37A—C36—C35	110.8 (4)	C75—C74—C73	119.18 (15)
C37A—C36—H36A	109.7	C75—C74—H74	120.4
C37A—C36—C43	105.1 (3)	C74—C75—H75	119.7
C43—C36—C35	111.71 (12)	C74—C75—C76	120.54 (15)
C43—C36—H36	104.8	C76—C75—H75	119.7
C43—C36—H36A	109.7	C71—C76—H76	119.6
C43—C36—C37	119.3 (3)	C75—C76—C71	120.88 (15)
C38—C37—C36	120.4 (3)	C75—C76—H76	119.6
C42—C37—C36	120.0 (6)	C58—C57—C50	113.5 (4)
C42—C37—C38	119.6 (6)	C58—C57—C62	119.6 (5)
C38A—C37A—C36	124.1 (5)	C62—C57—C50	126.7 (5)
			
Ni1—O1—C21—O2	11.3 (2)	C36—C37A—C42A—C41A	178.3 (6)
Ni1—O1—C21—C22	−167.06 (9)	C36—C43—C44—C45	173.6 (3)
Ni1—O3—C35—O4	6.9 (2)	C36—C43—C44A—C45A	−173.1 (3)
Ni1—O3—C35—C36	−173.47 (9)	C36—C43—C48—C47	178.30 (17)
Ni1—O5—C49—O6	0.3 (2)	C37—C36—C43—C44A	79.7 (4)
Ni1—O5—C49—C50	−178.68 (9)	C37—C36—C43—C48	−72.7 (4)
Ni1—N1—C1—C2	−179.68 (11)	C37—C38—C39—C40	−2.2 (8)
Ni1—N1—C5—C4	178.74 (11)	C37A—C36—C43—C44	123.1 (4)
Ni1—N2—C6—C7	−175.36 (11)	C37A—C36—C43—C48	−82.6 (4)
Ni1—N2—C10—C9	175.80 (12)	C37A—C38A—C39A—C40A	−1.2 (8)
Ni2—O4—C35—O3	9.4 (2)	C38—C37—C42—C41	−0.2 (9)
Ni2—O4—C35—C36	−170.28 (10)	C38—C39—C40—C41	0.8 (8)
Ni2—O6—C49—O5	13.1 (2)	C38A—C37A—C42A—C41A	2.4 (11)
Ni2—O6—C49—C50	−167.89 (10)	C38A—C39A—C40A—C41A	2.3 (9)
Ni2—O7—C63—O8	−17.3 (2)	C39—C40—C41—C42	0.9 (7)
Ni2—O7—C63—C64	163.81 (9)	C39A—C40A—C41A—C42A	−1.1 (10)
Ni2—N3—C11—C12	−172.46 (15)	C40—C41—C42—C37	−1.2 (7)
Ni2—N3—C15—C14	173.33 (13)	C40A—C41A—C42A—C37A	−1.3 (10)
Ni2—N4—C16—C17	177.54 (13)	C42—C37—C38—C39	1.9 (9)
Ni2—N4—C20—C19	−178.02 (15)	C42A—C37A—C38A—C39A	−1.1 (10)
O1—C21—C22—C23	−78.13 (16)	C43—C36—C37—C38	−50.1 (7)
O1—C21—C22—C29	49.23 (17)	C43—C36—C37—C42	132.1 (5)
O2—C21—C22—C23	103.38 (15)	C43—C36—C37A—C38A	−36.6 (8)
O2—C21—C22—C29	−129.26 (14)	C43—C36—C37A—C42A	147.8 (7)
O3—C35—C36—C37	128.5 (3)	C43—C44—C45—C46	−7.0 (6)
O3—C35—C36—C37A	146.5 (3)	C43—C44A—C45A—C46A	7.6 (5)
O3—C35—C36—C43	−96.69 (16)	C44—C43—C48—C47	−24.0 (3)
O4—C35—C36—C37	−51.8 (3)	C44—C45—C46—C47	1.9 (6)
O4—C35—C36—C37A	−33.8 (3)	C44A—C43—C48—C47	26.6 (3)
O4—C35—C36—C43	83.01 (17)	C44A—C45A—C46A—C47	−0.6 (6)
O5—C49—C50—C51	−71.40 (17)	C45—C46—C47—C48	−8.5 (5)
O5—C49—C50—C57A	64.0 (3)	C45A—C46A—C47—C48	5.1 (5)
O5—C49—C50—C57	45.3 (3)	C46—C47—C48—C43	21.7 (4)
O6—C49—C50—C51	109.49 (16)	C46A—C47—C48—C43	−18.7 (3)
O6—C49—C50—C57A	−115.1 (3)	C48—C43—C44—C45	17.0 (4)
O6—C49—C50—C57	−133.8 (3)	C48—C43—C44A—C45A	−19.9 (4)
O7—C63—C64—C65	−46.07 (17)	C49—C50—C51—C52	−166.44 (15)
O7—C63—C64—C71	81.84 (15)	C49—C50—C51—C56	13.3 (2)
O8—C63—C64—C65	134.96 (13)	C49—C50—C57A—C58A	61.4 (5)
O8—C63—C64—C71	−97.13 (15)	C49—C50—C57A—C62A	−116.0 (4)
N1—C1—C2—C3	1.2 (2)	C49—C50—C57—C58	66.8 (4)
N2—C6—C7—C8	−0.5 (2)	C49—C50—C57—C62	−108.3 (5)
N3—C11—C12—C13	−1.2 (3)	C50—C51—C52—C53	−178.3 (2)
N4—C16—C17—C18	0.7 (3)	C50—C51—C56—C55	177.42 (17)
C1—N1—C5—C4	−1.8 (2)	C50—C57A—C58A—C59A	−178.0 (4)
C1—C2—C3—C4	−2.3 (2)	C50—C57A—C62A—C61A	178.4 (4)
C2—C3—C4—C5	1.4 (2)	C51—C50—C57A—C58A	−170.2 (3)
C3—C4—C5—N1	0.7 (2)	C51—C50—C57A—C62A	12.4 (6)
C5—N1—C1—C2	0.9 (2)	C51—C50—C57—C58	−171.0 (3)
C6—N2—C10—C9	−0.9 (2)	C51—C50—C57—C62	14.0 (5)
C6—C7—C8—C9	−0.6 (2)	C51—C52—C53—C54	0.0 (4)
C7—C8—C9—C10	0.8 (2)	C52—C51—C56—C55	−2.8 (3)
C8—C9—C10—N2	−0.1 (3)	C52—C53—C54—C55	−1.2 (4)
C10—N2—C6—C7	1.2 (2)	C53—C54—C55—C56	0.3 (4)
C11—N3—C15—C14	−1.3 (2)	C54—C55—C56—C51	1.7 (3)
C11—C12—C13—C14	−1.2 (3)	C56—C51—C52—C53	2.0 (3)
C12—C13—C14—C15	2.2 (3)	C57A—C50—C51—C52	70.2 (3)
C13—C14—C15—N3	−1.0 (3)	C57A—C50—C51—C56	−110.1 (3)
C15—N3—C11—C12	2.4 (3)	C57A—C58A—C59A—C60A	−0.2 (6)
C16—N4—C20—C19	−0.5 (3)	C58—C59—C60—C61	0.3 (7)
C16—C17—C18—C19	−0.8 (3)	C58A—C57A—C62A—C61A	0.9 (7)
C17—C18—C19—C20	0.3 (3)	C58A—C59A—C60A—C61A	0.8 (8)
C18—C19—C20—N4	0.4 (3)	C59—C58—C57—C50	−174.3 (4)
C20—N4—C16—C17	0.0 (2)	C59—C58—C57—C62	1.2 (7)
C21—C22—C23—C24	−158.79 (13)	C59—C60—C61—C62	0.4 (7)
C21—C22—C23—C28	21.5 (2)	C59A—C60A—C61A—C62A	−0.5 (9)
C21—C22—C29—C30	79.85 (16)	C60—C61—C62—C57	−0.4 (6)
C21—C22—C29—C34	−97.64 (16)	C60A—C61A—C62A—C57A	−0.4 (8)
C22—C23—C24—C25	179.70 (15)	C61—C62—C57—C50	174.4 (4)
C22—C23—C28—C27	179.88 (14)	C61—C62—C57—C58	−0.4 (7)
C22—C29—C30—C31	−177.09 (13)	C62A—C57A—C58A—C59A	−0.6 (7)
C22—C29—C34—C33	176.92 (14)	C63—C64—C65—C66	91.85 (17)
C23—C22—C29—C30	−152.85 (13)	C63—C64—C65—C70	−89.17 (18)
C23—C22—C29—C34	29.7 (2)	C63—C64—C71—C72	130.31 (14)
C23—C24—C25—C26	0.7 (3)	C63—C64—C71—C76	−50.22 (18)
C24—C23—C28—C27	0.2 (2)	C64—C65—C66—C67	179.97 (16)
C24—C25—C26—C27	−0.4 (3)	C64—C65—C70—C69	178.8 (2)
C25—C26—C27—C28	0.0 (3)	C64—C71—C72—C73	177.80 (14)
C26—C27—C28—C23	0.1 (3)	C64—C71—C76—C75	−178.47 (14)
C28—C23—C24—C25	−0.6 (2)	C65—C64—C71—C72	−102.95 (16)
C29—C22—C23—C24	76.13 (17)	C65—C64—C71—C76	76.52 (17)
C29—C22—C23—C28	−103.56 (17)	C65—C66—C67—C68	1.1 (3)
C29—C30—C31—C32	0.0 (2)	C66—C65—C70—C69	−2.1 (3)
C30—C29—C34—C33	−0.6 (2)	C66—C67—C68—C69	−1.9 (3)
C30—C31—C32—C33	−0.3 (2)	C67—C68—C69—C70	0.8 (4)
C31—C32—C33—C34	0.2 (3)	C68—C69—C70—C65	1.3 (4)
C32—C33—C34—C29	0.2 (3)	C70—C65—C66—C67	1.0 (3)
C34—C29—C30—C31	0.5 (2)	C71—C64—C65—C66	−34.2 (2)
C35—C36—C37—C38	80.8 (6)	C71—C64—C65—C70	144.81 (16)
C35—C36—C37—C42	−97.0 (6)	C71—C72—C73—C74	0.9 (2)
C35—C36—C37A—C38A	84.2 (7)	C72—C71—C76—C75	1.0 (2)
C35—C36—C37A—C42A	−91.4 (8)	C72—C73—C74—C75	0.6 (2)
C35—C36—C43—C44	2.8 (3)	C73—C74—C75—C76	−1.3 (2)
C35—C36—C43—C44A	−50.5 (2)	C74—C75—C76—C71	0.5 (2)
C35—C36—C43—C48	157.13 (16)	C76—C71—C72—C73	−1.7 (2)
C36—C37—C38—C39	−175.9 (5)	C57—C50—C51—C52	71.8 (3)
C36—C37—C42—C41	177.6 (4)	C57—C50—C51—C56	−108.5 (3)
C36—C37A—C38A—C39A	−176.9 (5)	C57—C58—C59—C60	−1.1 (7)

### µ-Aqua-κ2O:O-di-µ-diphenylacetato-κ4O:O'-bis[(diphenylacetato-κO)bis(pyridine-κN)nickel(II)] (1) Hydrogen-bond geometry (Å, º)

**Table:**

*D*—H···*A*	*D*—H	H···*A*	*D*···*A*	*D*—H···*A*
O9—H9*A*···O2	0.85 (2)	1.79 (2)	2.6201 (15)	165 (2)
O9—H9*B*···O8	0.87 (3)	1.70 (3)	2.5617 (14)	169 (2)
O9—H9*B*···O7	0.87 (3)	2.60 (2)	3.0236 (14)	111.2 (18)
C1—H1···O5	0.93	2.52	2.9814 (18)	111
C5—H5···O1	0.93	2.51	2.9899 (18)	112
C11—H11···O7	0.93	2.45	2.9850 (19)	117
C8—H8···O8^i^	0.93	2.39	3.211 (2)	148
C12—H12···O2^ii^	0.93	2.46	3.302 (2)	151
C15—H15···O4	0.93	2.60	3.089 (2)	113

Symmetry codes: (i) −*x*+2, −*y*+1, −*z*+1; (ii) −*x*+1, −*y*+1, −*z*+1.

### µ-Aqua-κ2O:O-di-µ-diphenylacetato-κ4O:O'-\ bis[(2,2'-bipyridine-κ2N,N')(diphenylacetato-κO)\ nickel(II)]–acetonitrile–diphenylacetic acid (1/2.5/1) (2) Crystal data

**Table:**

[Ni_2_(C_14_H_11_O_2_)_4_(C_10_H_8_N_2_)_2_(H_2_O)]·2.5C_2_H_3_N·C_14_H_12_O_2_	*Z* = 2
*M**_r_* = 1607.58	*F*(000) = 1682
Triclinic, *P*1	*D*_x_ = 1.301 Mg m^−^^3^
*a* = 15.6064 (3) Å	Cu *K*α radiation, λ = 1.54184 Å
*b* = 17.7440 (4) Å	Cell parameters from 31385 reflections
*c* = 17.9143 (4) Å	θ = 2.6–71.7°
α = 70.462 (2)°	µ = 1.10 mm^−^^1^
β = 69.631 (2)°	*T* = 100 K
γ = 64.726 (2)°	Prism, light green
*V* = 4103.17 (18) Å^3^	0.22 × 0.14 × 0.08 mm

### µ-Aqua-κ2O:O-di-µ-diphenylacetato-κ4O:O'-\ bis[(2,2'-bipyridine-κ2N,N')(diphenylacetato-κO)\ nickel(II)]–acetonitrile–diphenylacetic acid (1/2.5/1) (2) Data collection

**Table:**

Rigaku SuperNova, Single source at offset/far, HyPix3000 diffractometer	15557 independent reflections
Radiation source: micro-focus sealed X-ray tube, SuperNova (Cu) X-ray Source	13874 reflections with *I* > 2σ(*I*)
Mirror monochromator	*R*_int_ = 0.045
ω scans	θ_max_ = 70.0°, θ_min_ = 2.7°
Absorption correction: multi-scan (CrysAlis PRO; Rigaku OD, 2017)	*h* = −19→18
*T*_min_ = 0.809, *T*_max_ = 1.000	*k* = −21→21
52698 measured reflections	*l* = −21→21

### µ-Aqua-κ2O:O-di-µ-diphenylacetato-κ4O:O'-\ bis[(2,2'-bipyridine-κ2N,N')(diphenylacetato-κO)\ nickel(II)]–acetonitrile–diphenylacetic acid (1/2.5/1) (2) Refinement

**Table:**

Refinement on *F*^2^	Primary atom site location: dual
Least-squares matrix: full	Secondary atom site location: difference Fourier map
*R*[*F*^2^ > 2σ(*F*^2^)] = 0.038	Hydrogen site location: mixed
*wR*(*F*^2^) = 0.103	H atoms treated by a mixture of independent and constrained refinement
*S* = 1.06	*w* = 1/[σ^2^(*F*_o_^2^) + (0.0489*P*)^2^ + 1.9174*P*] where *P* = (*F*_o_^2^ + 2*F*_c_^2^)/3
15557 reflections	(Δ/σ)_max_ < 0.001
1079 parameters	Δρ_max_ = 0.48 e Å^−^^3^
1 restraint	Δρ_min_ = −0.57 e Å^−^^3^

### µ-Aqua-κ2O:O-di-µ-diphenylacetato-κ4O:O'-\ bis[(2,2'-bipyridine-κ2N,N')(diphenylacetato-κO)\ nickel(II)]–acetonitrile–diphenylacetic acid (1/2.5/1) (2) Special details

**Table:**

Geometry. All esds (except the esd in the dihedral angle between two l.s. planes) are estimated using the full covariance matrix. The cell esds are taken into account individually in the estimation of esds in distances, angles and torsion angles; correlations between esds in cell parameters are only used when they are defined by crystal symmetry. An approximate (isotropic) treatment of cell esds is used for estimating esds involving l.s. planes.

### µ-Aqua-κ2O:O-di-µ-diphenylacetato-κ4O:O'-\ bis[(2,2'-bipyridine-κ2N,N')(diphenylacetato-κO)\ nickel(II)]–acetonitrile–diphenylacetic acid (1/2.5/1) (2) Fractional atomic coordinates and isotropic or equivalent isotropic displacement parameters (Å^2^)

**Table:**

	*x*	*y*	*z*	*U*_iso_*/*U*_eq_	Occ. (<1)
Ni1	0.48008 (2)	0.65582 (2)	0.79941 (2)	0.01430 (7)	
Ni2	0.53556 (2)	0.83920 (2)	0.69046 (2)	0.01383 (7)	
O1	0.49052 (8)	0.60373 (7)	0.70516 (7)	0.0185 (2)	
O2	0.63673 (8)	0.61134 (7)	0.62971 (7)	0.0198 (2)	
O3	0.36089 (8)	0.75365 (7)	0.76627 (7)	0.0175 (2)	
O4	0.41490 (8)	0.84939 (7)	0.66178 (7)	0.0179 (2)	
O5	0.46560 (8)	0.71448 (7)	0.88737 (6)	0.0175 (2)	
O6	0.46012 (8)	0.84528 (7)	0.80835 (6)	0.0183 (2)	
O7	0.65406 (8)	0.83659 (7)	0.71911 (7)	0.0194 (2)	
O8	0.73202 (8)	0.70008 (7)	0.77037 (7)	0.0223 (2)	
O9	0.58547 (8)	0.70752 (7)	0.72274 (7)	0.0152 (2)	
H9A	0.6353 (19)	0.7009 (16)	0.7424 (15)	0.048 (7)*	
H9B	0.612 (2)	0.6715 (17)	0.6843 (17)	0.056 (8)*	
N1	0.58608 (10)	0.53971 (8)	0.84176 (8)	0.0165 (3)	
N2	0.39298 (10)	0.58745 (8)	0.87998 (8)	0.0172 (3)	
N3	0.50578 (9)	0.96934 (8)	0.65646 (8)	0.0155 (3)	
N4	0.61480 (9)	0.85119 (8)	0.56857 (8)	0.0160 (3)	
C1	0.68338 (12)	0.51896 (10)	0.81861 (10)	0.0190 (3)	
H1	0.708989	0.559389	0.780761	0.023*	
C2	0.74780 (12)	0.43986 (11)	0.84850 (10)	0.0226 (3)	
H2	0.814957	0.427065	0.830264	0.027*	
C3	0.70891 (13)	0.38054 (10)	0.90639 (11)	0.0239 (4)	
H3	0.749950	0.327487	0.928423	0.029*	
C4	0.60855 (13)	0.40093 (10)	0.93110 (10)	0.0213 (3)	
H4	0.581449	0.361985	0.970044	0.026*	
C5	0.54871 (12)	0.48098 (10)	0.89654 (9)	0.0179 (3)	
C6	0.44044 (12)	0.50713 (10)	0.91752 (9)	0.0175 (3)	
C7	0.39047 (13)	0.45370 (11)	0.97104 (10)	0.0225 (3)	
H7	0.424302	0.398764	0.996237	0.027*	
C8	0.28924 (13)	0.48339 (11)	0.98648 (11)	0.0250 (4)	
H8	0.254386	0.448614	1.022042	0.030*	
C9	0.24107 (13)	0.56570 (11)	0.94800 (10)	0.0247 (4)	
H9	0.173410	0.586946	0.957138	0.030*	
C10	0.29551 (12)	0.61580 (11)	0.89564 (10)	0.0209 (3)	
H10	0.262997	0.671230	0.870454	0.025*	
C11	0.45404 (12)	1.02389 (10)	0.70602 (10)	0.0197 (3)	
H11	0.422318	1.004061	0.758871	0.024*	
C12	0.44597 (12)	1.10901 (11)	0.68143 (11)	0.0227 (3)	
H12	0.409581	1.145597	0.717214	0.027*	
C13	0.49305 (12)	1.13866 (10)	0.60261 (11)	0.0228 (3)	
H13	0.488382	1.195575	0.584650	0.027*	
C14	0.54719 (12)	1.08241 (10)	0.55088 (10)	0.0198 (3)	
H14	0.579208	1.100977	0.497704	0.024*	
C15	0.55274 (11)	0.99750 (10)	0.58005 (10)	0.0165 (3)	
C16	0.61181 (11)	0.93211 (10)	0.53012 (9)	0.0158 (3)	
C17	0.66203 (12)	0.95145 (10)	0.44910 (10)	0.0194 (3)	
H17	0.656395	1.007695	0.422863	0.023*	
C18	0.72045 (12)	0.88599 (11)	0.40804 (10)	0.0219 (3)	
H18	0.755948	0.897484	0.354473	0.026*	
C19	0.72511 (12)	0.80302 (11)	0.44818 (10)	0.0220 (3)	
H19	0.764480	0.757818	0.422320	0.026*	
C20	0.66994 (12)	0.78890 (10)	0.52753 (10)	0.0194 (3)	
H20	0.671250	0.733565	0.553528	0.023*	
C21	0.35006 (11)	0.81917 (10)	0.70956 (9)	0.0157 (3)	
C22	0.24624 (11)	0.87016 (10)	0.69508 (10)	0.0175 (3)	
H22	0.232261	0.929763	0.694177	0.021*	
C23	0.24589 (11)	0.86855 (10)	0.61060 (10)	0.0174 (3)	
C24	0.29523 (12)	0.79379 (10)	0.58092 (10)	0.0206 (3)	
H24	0.329078	0.743903	0.613017	0.025*	
C25	0.29421 (12)	0.79327 (11)	0.50377 (11)	0.0241 (4)	
H25	0.326012	0.742855	0.485038	0.029*	
C26	0.24560 (12)	0.86817 (12)	0.45449 (10)	0.0245 (4)	
H26	0.245433	0.867904	0.402720	0.029*	
C27	0.19759 (12)	0.94294 (11)	0.48275 (10)	0.0233 (3)	
H27	0.165635	0.993117	0.449745	0.028*	
C28	0.19710 (11)	0.94312 (10)	0.56054 (10)	0.0193 (3)	
H28	0.163930	0.993413	0.579447	0.023*	
C29	0.16779 (11)	0.84258 (10)	0.76417 (10)	0.0198 (3)	
C30	0.12384 (15)	0.88219 (14)	0.82986 (12)	0.0346 (4)	
H30	0.142474	0.925150	0.830629	0.042*	
C31	0.05241 (18)	0.85864 (17)	0.89449 (13)	0.0486 (6)	
H31	0.023242	0.886268	0.937709	0.058*	
C32	0.02449 (15)	0.79395 (15)	0.89470 (12)	0.0364 (5)	
H32	−0.023219	0.778066	0.937905	0.044*	
C33	0.06808 (13)	0.75360 (13)	0.83038 (12)	0.0305 (4)	
H33	0.050244	0.709887	0.830271	0.037*	
C34	0.13897 (13)	0.77814 (11)	0.76533 (11)	0.0262 (4)	
H34	0.167465	0.750807	0.721927	0.031*	
C35	0.44896 (11)	0.79360 (10)	0.87518 (9)	0.0164 (3)	
C36	0.41004 (12)	0.82922 (10)	0.95331 (9)	0.0185 (3)	
H36	0.354080	0.811623	0.986419	0.022*	
C37	0.48648 (12)	0.78748 (10)	1.00317 (10)	0.0190 (3)	
C38	0.45875 (14)	0.76057 (11)	1.08758 (10)	0.0243 (4)	
H38	0.393679	0.767059	1.112933	0.029*	
C39	0.52729 (15)	0.72404 (12)	1.13450 (11)	0.0308 (4)	
H39	0.507869	0.706461	1.190834	0.037*	
C40	0.62440 (15)	0.71393 (11)	1.09719 (12)	0.0295 (4)	
H40	0.670004	0.690449	1.128580	0.035*	
C41	0.65370 (14)	0.73885 (11)	1.01294 (12)	0.0279 (4)	
H41	0.719122	0.730931	0.987748	0.033*	
C42	0.58490 (13)	0.77572 (11)	0.96624 (10)	0.0232 (3)	
H42	0.604697	0.792697	0.909870	0.028*	
C43	0.37201 (13)	0.92683 (10)	0.93423 (10)	0.0218 (3)	
C44	0.27937 (14)	0.97071 (12)	0.91935 (11)	0.0289 (4)	
H44	0.241471	0.940156	0.923569	0.035*	
C45	0.24237 (16)	1.05974 (13)	0.89821 (12)	0.0372 (5)	
H45	0.180404	1.088070	0.888234	0.045*	
C46	0.29814 (17)	1.10600 (12)	0.89207 (12)	0.0366 (5)	
H46	0.273687	1.165372	0.878082	0.044*	
C47	0.39007 (16)	1.06340 (12)	0.90685 (12)	0.0329 (4)	
H47	0.427576	1.094212	0.902899	0.039*	
C48	0.42706 (14)	0.97426 (11)	0.92772 (11)	0.0262 (4)	
H48	0.489187	0.946202	0.937382	0.031*	
C49	0.56412 (11)	0.58864 (10)	0.64647 (9)	0.0174 (3)	
C50	0.57011 (12)	0.53499 (10)	0.59164 (10)	0.0192 (3)	
H50	0.508387	0.524739	0.609162	0.023*	
C51	0.58260 (12)	0.58163 (10)	0.50230 (10)	0.0195 (3)	
C52	0.51097 (13)	0.60167 (10)	0.46238 (11)	0.0225 (3)	
H52	0.455630	0.587378	0.490997	0.027*	
C53	0.52074 (14)	0.64282 (11)	0.38019 (11)	0.0260 (4)	
H53	0.471809	0.656302	0.354592	0.031*	
C54	0.60332 (14)	0.66364 (11)	0.33663 (11)	0.0273 (4)	
H54	0.610502	0.690168	0.281556	0.033*	
C55	0.67510 (13)	0.64478 (11)	0.37547 (11)	0.0262 (4)	
H55	0.730455	0.658928	0.346437	0.031*	
C56	0.66473 (12)	0.60474 (11)	0.45774 (10)	0.0231 (3)	
H56	0.712837	0.593173	0.483497	0.028*	
C57	0.65133 (13)	0.44844 (10)	0.60972 (10)	0.0225 (3)	
C58	0.64922 (15)	0.40498 (11)	0.69087 (11)	0.0293 (4)	
H58	0.598061	0.427982	0.732212	0.035*	
C59	0.72236 (18)	0.32799 (12)	0.71066 (13)	0.0394 (5)	
H59	0.720099	0.299903	0.765039	0.047*	
C60	0.79861 (19)	0.29282 (13)	0.64989 (14)	0.0468 (6)	
H60	0.848441	0.241750	0.663116	0.056*	
C61	0.80003 (19)	0.33431 (14)	0.56931 (14)	0.0488 (6)	
H61	0.850476	0.310422	0.528070	0.059*	
C62	0.72678 (16)	0.41137 (12)	0.54935 (12)	0.0350 (4)	
H62	0.728404	0.438390	0.494809	0.042*	
C63	0.72383 (11)	0.77861 (10)	0.74597 (9)	0.0169 (3)	
C64	0.80523 (11)	0.80416 (10)	0.74729 (10)	0.0182 (3)	
H64	0.842930	0.758017	0.784632	0.022*	
C65	0.87439 (11)	0.81350 (10)	0.66271 (10)	0.0180 (3)	
C66	0.95957 (13)	0.82589 (12)	0.65567 (11)	0.0263 (4)	
H66	0.972867	0.826310	0.702232	0.032*	
C67	1.02485 (13)	0.83759 (12)	0.58085 (12)	0.0299 (4)	
H67	1.081118	0.845864	0.577599	0.036*	
C68	1.00631 (12)	0.83695 (11)	0.51108 (11)	0.0247 (4)	
H68	1.049263	0.845778	0.460590	0.030*	
C69	0.92303 (13)	0.82302 (12)	0.51719 (11)	0.0285 (4)	
H69	0.910804	0.821342	0.470638	0.034*	
C70	0.85750 (12)	0.81150 (12)	0.59241 (11)	0.0261 (4)	
H70	0.801798	0.802374	0.595575	0.031*	
C71	0.76376 (11)	0.88550 (11)	0.77937 (10)	0.0197 (3)	
C72	0.70963 (12)	0.96398 (11)	0.73765 (11)	0.0228 (3)	
H72	0.700113	0.967920	0.687836	0.027*	
C73	0.66983 (14)	1.03629 (12)	0.77002 (13)	0.0310 (4)	
H73	0.632863	1.088071	0.742332	0.037*	
C74	0.68524 (15)	1.03124 (14)	0.84358 (13)	0.0379 (5)	
H74	0.658301	1.079553	0.865327	0.045*	
C75	0.74056 (16)	0.95449 (14)	0.88447 (12)	0.0378 (5)	
H75	0.751926	0.951382	0.933229	0.045*	
C76	0.77938 (14)	0.88169 (13)	0.85280 (11)	0.0276 (4)	
H76	0.816069	0.830049	0.880898	0.033*	
O10	0.89816 (10)	0.61438 (9)	0.89341 (9)	0.0355 (3)	
O11	0.88935 (9)	0.57424 (8)	0.79119 (8)	0.0289 (3)	
H11A	0.838938	0.616186	0.792339	0.043*	
C77	0.93148 (12)	0.56702 (11)	0.84692 (11)	0.0237 (4)	
C78	1.02974 (12)	0.49329 (11)	0.84603 (11)	0.0235 (3)	
H78	1.039539	0.476404	0.901272	0.028*	
C79	1.03147 (12)	0.41411 (11)	0.82837 (11)	0.0237 (4)	
C80	1.05206 (13)	0.40451 (11)	0.74937 (11)	0.0264 (4)	
H80	1.066850	0.446779	0.705243	0.032*	
C81	1.05078 (14)	0.33216 (12)	0.73555 (12)	0.0305 (4)	
H81	1.064899	0.326481	0.682382	0.037*	
C82	1.02850 (16)	0.26856 (12)	0.80082 (13)	0.0359 (4)	
H82	1.027416	0.220442	0.791612	0.043*	
C83	1.00797 (17)	0.27756 (13)	0.87961 (13)	0.0401 (5)	
H83	0.992834	0.235336	0.923606	0.048*	
C84	1.00983 (15)	0.34950 (12)	0.89346 (12)	0.0319 (4)	
H84	0.996505	0.354561	0.946720	0.038*	
C85	1.11269 (12)	0.52450 (11)	0.78942 (11)	0.0242 (4)	
C86	1.10475 (14)	0.58408 (12)	0.71570 (11)	0.0292 (4)	
H86	1.046494	0.607358	0.699466	0.035*	
C87	1.18377 (15)	0.60882 (13)	0.66634 (13)	0.0350 (4)	
H87	1.177694	0.648958	0.617503	0.042*	
C88	1.27130 (15)	0.57424 (13)	0.68924 (14)	0.0386 (5)	
H88	1.324112	0.590409	0.655647	0.046*	
C89	1.27958 (14)	0.51551 (13)	0.76243 (15)	0.0385 (5)	
H89	1.338045	0.492216	0.778348	0.046*	
C90	1.20077 (13)	0.49130 (12)	0.81212 (13)	0.0302 (4)	
H90	1.206847	0.452185	0.861503	0.036*	
N6	0.89316 (15)	0.60085 (15)	0.47738 (14)	0.0648 (7)	
C93	0.88350 (15)	0.55719 (16)	0.54003 (14)	0.0442 (5)	
C94	0.87032 (16)	0.50006 (17)	0.62001 (14)	0.0516 (6)	
H94A	0.916505	0.443505	0.616880	0.077*	
H94B	0.805227	0.499029	0.637100	0.077*	
H94C	0.880535	0.520018	0.658735	0.077*	
N7	0.0085 (7)	0.9325 (6)	1.0732 (5)	0.048 (2)	0.25
N7A	0.2022 (7)	0.8257 (6)	1.1303 (6)	0.054 (2)	0.25
C95	0.0792 (9)	0.9005 (6)	1.0992 (6)	0.029 (2)	0.25
C95A	0.1436 (11)	0.8766 (8)	1.1074 (6)	0.032 (3)	0.25
C96	0.1568 (13)	0.8685 (14)	1.1297 (10)	0.077 (5)	0.25
H96A	0.211175	0.833762	1.095444	0.116*	0.25
H96B	0.143095	0.834327	1.183626	0.116*	0.25
H96C	0.172317	0.914338	1.132223	0.116*	0.25
C96A	0.0590 (11)	0.9333 (12)	1.0781 (9)	0.077 (5)	0.25
H96D	0.077731	0.953874	1.020192	0.116*	0.25
H96E	0.026954	0.980730	1.104414	0.116*	0.25
H96F	0.015204	0.903620	1.089938	0.116*	0.25
N5	0.03424 (17)	1.10131 (13)	0.70838 (13)	0.0529 (5)	
C91	−0.00598 (16)	1.06094 (13)	0.70844 (14)	0.0394 (5)	
C92	−0.05605 (17)	1.00871 (16)	0.70753 (17)	0.0521 (6)	
H92A	−0.033938	0.994942	0.654951	0.078*	
H92B	−0.125133	1.039643	0.718338	0.078*	
H92C	−0.042191	0.956998	0.748700	0.078*	

### µ-Aqua-κ2O:O-di-µ-diphenylacetato-κ4O:O'-\ bis[(2,2'-bipyridine-κ2N,N')(diphenylacetato-κO)\ nickel(II)]–acetonitrile–diphenylacetic acid (1/2.5/1) (2) Atomic displacement parameters (Å^2^)

**Table:**

	*U*^11^	*U*^22^	*U*^33^	*U*^12^	*U*^13^	*U*^23^
Ni1	0.01608 (13)	0.01387 (13)	0.01361 (13)	−0.00694 (10)	−0.00404 (10)	−0.00111 (10)
Ni2	0.01495 (13)	0.01422 (13)	0.01300 (13)	−0.00682 (10)	−0.00418 (10)	−0.00069 (10)
O1	0.0194 (5)	0.0220 (6)	0.0164 (5)	−0.0093 (5)	−0.0027 (4)	−0.0058 (4)
O2	0.0202 (6)	0.0225 (6)	0.0201 (6)	−0.0103 (5)	−0.0023 (4)	−0.0077 (5)
O3	0.0173 (5)	0.0165 (5)	0.0179 (5)	−0.0074 (4)	−0.0050 (4)	0.0000 (4)
O4	0.0169 (5)	0.0210 (5)	0.0171 (5)	−0.0103 (4)	−0.0056 (4)	0.0006 (4)
O5	0.0235 (6)	0.0160 (5)	0.0147 (5)	−0.0089 (4)	−0.0053 (4)	−0.0020 (4)
O6	0.0230 (6)	0.0167 (5)	0.0144 (5)	−0.0089 (4)	−0.0027 (4)	−0.0019 (4)
O7	0.0190 (5)	0.0175 (5)	0.0244 (6)	−0.0077 (4)	−0.0106 (5)	−0.0006 (4)
O8	0.0207 (6)	0.0166 (5)	0.0318 (7)	−0.0083 (5)	−0.0115 (5)	−0.0004 (5)
O9	0.0169 (5)	0.0147 (5)	0.0155 (5)	−0.0070 (4)	−0.0048 (4)	−0.0025 (4)
N1	0.0202 (7)	0.0165 (6)	0.0151 (6)	−0.0082 (5)	−0.0053 (5)	−0.0029 (5)
N2	0.0213 (7)	0.0181 (6)	0.0140 (6)	−0.0100 (5)	−0.0032 (5)	−0.0028 (5)
N3	0.0157 (6)	0.0159 (6)	0.0157 (6)	−0.0069 (5)	−0.0061 (5)	−0.0001 (5)
N4	0.0159 (6)	0.0173 (6)	0.0158 (6)	−0.0075 (5)	−0.0055 (5)	−0.0009 (5)
C1	0.0210 (8)	0.0186 (8)	0.0189 (8)	−0.0089 (6)	−0.0060 (6)	−0.0021 (6)
C2	0.0214 (8)	0.0225 (8)	0.0241 (9)	−0.0069 (7)	−0.0073 (7)	−0.0045 (7)
C3	0.0275 (9)	0.0163 (8)	0.0264 (9)	−0.0039 (7)	−0.0119 (7)	−0.0022 (7)
C4	0.0285 (9)	0.0174 (8)	0.0179 (8)	−0.0098 (7)	−0.0064 (7)	−0.0005 (6)
C5	0.0241 (8)	0.0171 (7)	0.0150 (7)	−0.0099 (6)	−0.0041 (6)	−0.0037 (6)
C6	0.0231 (8)	0.0162 (7)	0.0149 (7)	−0.0087 (6)	−0.0032 (6)	−0.0042 (6)
C7	0.0283 (9)	0.0186 (8)	0.0194 (8)	−0.0116 (7)	−0.0015 (7)	−0.0028 (6)
C8	0.0291 (9)	0.0249 (8)	0.0222 (8)	−0.0180 (7)	0.0026 (7)	−0.0047 (7)
C9	0.0226 (8)	0.0287 (9)	0.0227 (9)	−0.0127 (7)	−0.0006 (7)	−0.0058 (7)
C10	0.0205 (8)	0.0219 (8)	0.0200 (8)	−0.0088 (7)	−0.0036 (6)	−0.0040 (6)
C11	0.0200 (8)	0.0196 (8)	0.0192 (8)	−0.0070 (6)	−0.0051 (6)	−0.0035 (6)
C12	0.0243 (8)	0.0192 (8)	0.0242 (9)	−0.0070 (7)	−0.0055 (7)	−0.0054 (7)
C13	0.0238 (8)	0.0155 (7)	0.0288 (9)	−0.0080 (6)	−0.0088 (7)	−0.0006 (7)
C14	0.0188 (8)	0.0197 (8)	0.0207 (8)	−0.0096 (6)	−0.0051 (6)	−0.0001 (6)
C15	0.0144 (7)	0.0177 (7)	0.0188 (8)	−0.0075 (6)	−0.0074 (6)	0.0005 (6)
C16	0.0148 (7)	0.0180 (7)	0.0167 (7)	−0.0079 (6)	−0.0069 (6)	−0.0002 (6)
C17	0.0204 (8)	0.0215 (8)	0.0188 (8)	−0.0117 (6)	−0.0070 (6)	0.0011 (6)
C18	0.0216 (8)	0.0286 (9)	0.0160 (8)	−0.0128 (7)	−0.0030 (6)	−0.0020 (7)
C19	0.0230 (8)	0.0241 (8)	0.0189 (8)	−0.0093 (7)	−0.0034 (6)	−0.0053 (6)
C20	0.0220 (8)	0.0179 (7)	0.0194 (8)	−0.0080 (6)	−0.0062 (6)	−0.0026 (6)
C21	0.0179 (7)	0.0169 (7)	0.0155 (7)	−0.0079 (6)	−0.0055 (6)	−0.0036 (6)
C22	0.0176 (7)	0.0166 (7)	0.0196 (8)	−0.0075 (6)	−0.0063 (6)	−0.0017 (6)
C23	0.0147 (7)	0.0211 (8)	0.0188 (8)	−0.0101 (6)	−0.0054 (6)	−0.0008 (6)
C24	0.0189 (8)	0.0208 (8)	0.0231 (8)	−0.0094 (6)	−0.0057 (6)	−0.0020 (6)
C25	0.0209 (8)	0.0283 (9)	0.0267 (9)	−0.0115 (7)	−0.0022 (7)	−0.0102 (7)
C26	0.0230 (8)	0.0376 (10)	0.0178 (8)	−0.0162 (7)	−0.0046 (7)	−0.0049 (7)
C27	0.0202 (8)	0.0291 (9)	0.0207 (8)	−0.0118 (7)	−0.0084 (7)	0.0024 (7)
C28	0.0158 (7)	0.0210 (8)	0.0218 (8)	−0.0087 (6)	−0.0051 (6)	−0.0019 (6)
C29	0.0170 (7)	0.0224 (8)	0.0180 (8)	−0.0075 (6)	−0.0080 (6)	0.0027 (6)
C30	0.0400 (11)	0.0499 (12)	0.0236 (9)	−0.0289 (10)	−0.0012 (8)	−0.0089 (8)
C31	0.0562 (14)	0.0766 (17)	0.0221 (10)	−0.0419 (13)	0.0076 (10)	−0.0149 (11)
C32	0.0318 (10)	0.0579 (13)	0.0207 (9)	−0.0283 (10)	−0.0051 (8)	0.0050 (9)
C33	0.0267 (9)	0.0360 (10)	0.0311 (10)	−0.0200 (8)	−0.0110 (8)	0.0060 (8)
C34	0.0239 (9)	0.0279 (9)	0.0284 (9)	−0.0135 (7)	−0.0043 (7)	−0.0042 (7)
C35	0.0157 (7)	0.0183 (7)	0.0167 (8)	−0.0075 (6)	−0.0042 (6)	−0.0032 (6)
C36	0.0221 (8)	0.0180 (8)	0.0151 (8)	−0.0088 (6)	−0.0027 (6)	−0.0027 (6)
C37	0.0274 (8)	0.0145 (7)	0.0171 (8)	−0.0077 (6)	−0.0066 (6)	−0.0041 (6)
C38	0.0321 (9)	0.0227 (8)	0.0178 (8)	−0.0092 (7)	−0.0056 (7)	−0.0051 (7)
C39	0.0463 (11)	0.0283 (9)	0.0184 (9)	−0.0110 (8)	−0.0134 (8)	−0.0027 (7)
C40	0.0412 (11)	0.0225 (9)	0.0305 (10)	−0.0076 (8)	−0.0209 (8)	−0.0045 (7)
C41	0.0293 (9)	0.0250 (9)	0.0337 (10)	−0.0099 (7)	−0.0120 (8)	−0.0065 (7)
C42	0.0287 (9)	0.0227 (8)	0.0199 (8)	−0.0114 (7)	−0.0058 (7)	−0.0036 (7)
C43	0.0292 (9)	0.0203 (8)	0.0131 (7)	−0.0076 (7)	−0.0014 (6)	−0.0055 (6)
C44	0.0304 (9)	0.0270 (9)	0.0265 (9)	−0.0061 (8)	−0.0075 (7)	−0.0072 (7)
C45	0.0365 (11)	0.0292 (10)	0.0337 (11)	0.0026 (8)	−0.0115 (9)	−0.0080 (8)
C46	0.0537 (13)	0.0176 (8)	0.0282 (10)	−0.0048 (8)	−0.0068 (9)	−0.0060 (7)
C47	0.0486 (12)	0.0230 (9)	0.0270 (10)	−0.0150 (8)	−0.0038 (8)	−0.0076 (7)
C48	0.0345 (10)	0.0221 (8)	0.0218 (9)	−0.0100 (7)	−0.0055 (7)	−0.0058 (7)
C49	0.0199 (8)	0.0156 (7)	0.0167 (8)	−0.0060 (6)	−0.0061 (6)	−0.0018 (6)
C50	0.0211 (8)	0.0206 (8)	0.0196 (8)	−0.0099 (6)	−0.0040 (6)	−0.0064 (6)
C51	0.0234 (8)	0.0178 (7)	0.0198 (8)	−0.0064 (6)	−0.0048 (6)	−0.0085 (6)
C52	0.0263 (8)	0.0196 (8)	0.0253 (9)	−0.0082 (7)	−0.0076 (7)	−0.0075 (7)
C53	0.0362 (10)	0.0219 (8)	0.0260 (9)	−0.0092 (7)	−0.0159 (8)	−0.0051 (7)
C54	0.0429 (11)	0.0242 (8)	0.0179 (8)	−0.0143 (8)	−0.0076 (7)	−0.0047 (7)
C55	0.0302 (9)	0.0287 (9)	0.0211 (9)	−0.0141 (7)	0.0003 (7)	−0.0088 (7)
C56	0.0233 (8)	0.0263 (8)	0.0223 (8)	−0.0090 (7)	−0.0051 (7)	−0.0085 (7)
C57	0.0306 (9)	0.0183 (8)	0.0236 (8)	−0.0098 (7)	−0.0087 (7)	−0.0069 (7)
C58	0.0405 (10)	0.0224 (9)	0.0243 (9)	−0.0118 (8)	−0.0053 (8)	−0.0062 (7)
C59	0.0626 (14)	0.0239 (9)	0.0276 (10)	−0.0103 (9)	−0.0167 (10)	−0.0014 (8)
C60	0.0593 (14)	0.0245 (10)	0.0405 (12)	0.0073 (10)	−0.0213 (11)	−0.0075 (9)
C61	0.0553 (14)	0.0349 (11)	0.0337 (11)	0.0081 (10)	−0.0089 (10)	−0.0141 (9)
C62	0.0437 (11)	0.0268 (9)	0.0247 (9)	−0.0006 (8)	−0.0091 (8)	−0.0085 (8)
C63	0.0168 (7)	0.0193 (8)	0.0151 (7)	−0.0072 (6)	−0.0029 (6)	−0.0041 (6)
C64	0.0181 (7)	0.0189 (7)	0.0197 (8)	−0.0082 (6)	−0.0079 (6)	−0.0010 (6)
C65	0.0156 (7)	0.0150 (7)	0.0233 (8)	−0.0050 (6)	−0.0058 (6)	−0.0034 (6)
C66	0.0229 (8)	0.0374 (10)	0.0250 (9)	−0.0161 (8)	−0.0045 (7)	−0.0086 (7)
C67	0.0209 (8)	0.0395 (10)	0.0348 (10)	−0.0173 (8)	−0.0011 (7)	−0.0116 (8)
C68	0.0203 (8)	0.0233 (8)	0.0249 (9)	−0.0067 (7)	0.0005 (7)	−0.0058 (7)
C69	0.0234 (9)	0.0392 (10)	0.0231 (9)	−0.0093 (8)	−0.0050 (7)	−0.0101 (8)
C70	0.0189 (8)	0.0379 (10)	0.0262 (9)	−0.0131 (7)	−0.0043 (7)	−0.0095 (8)
C71	0.0172 (7)	0.0248 (8)	0.0213 (8)	−0.0132 (7)	−0.0014 (6)	−0.0058 (7)
C72	0.0223 (8)	0.0245 (8)	0.0254 (9)	−0.0124 (7)	−0.0050 (7)	−0.0054 (7)
C73	0.0256 (9)	0.0264 (9)	0.0425 (11)	−0.0101 (7)	−0.0034 (8)	−0.0132 (8)
C74	0.0390 (11)	0.0393 (11)	0.0407 (12)	−0.0188 (9)	0.0053 (9)	−0.0242 (9)
C75	0.0474 (12)	0.0535 (13)	0.0244 (10)	−0.0271 (10)	−0.0008 (9)	−0.0185 (9)
C76	0.0306 (9)	0.0375 (10)	0.0214 (9)	−0.0193 (8)	−0.0051 (7)	−0.0058 (7)
O10	0.0316 (7)	0.0343 (7)	0.0369 (8)	−0.0010 (6)	−0.0093 (6)	−0.0161 (6)
O11	0.0232 (6)	0.0235 (6)	0.0415 (8)	−0.0022 (5)	−0.0148 (6)	−0.0095 (5)
C77	0.0214 (8)	0.0222 (8)	0.0261 (9)	−0.0084 (7)	−0.0054 (7)	−0.0028 (7)
C78	0.0211 (8)	0.0244 (8)	0.0237 (9)	−0.0051 (7)	−0.0075 (7)	−0.0053 (7)
C79	0.0181 (8)	0.0222 (8)	0.0269 (9)	−0.0037 (7)	−0.0068 (7)	−0.0037 (7)
C80	0.0241 (9)	0.0255 (9)	0.0262 (9)	−0.0077 (7)	−0.0059 (7)	−0.0030 (7)
C81	0.0299 (9)	0.0303 (9)	0.0299 (10)	−0.0069 (8)	−0.0082 (8)	−0.0090 (8)
C82	0.0430 (11)	0.0239 (9)	0.0410 (11)	−0.0087 (8)	−0.0137 (9)	−0.0078 (8)
C83	0.0562 (13)	0.0251 (10)	0.0330 (11)	−0.0148 (9)	−0.0115 (10)	0.0021 (8)
C84	0.0378 (10)	0.0256 (9)	0.0252 (9)	−0.0064 (8)	−0.0075 (8)	−0.0034 (7)
C85	0.0215 (8)	0.0240 (8)	0.0281 (9)	−0.0059 (7)	−0.0047 (7)	−0.0108 (7)
C86	0.0277 (9)	0.0304 (9)	0.0288 (10)	−0.0099 (8)	−0.0060 (7)	−0.0069 (8)
C87	0.0390 (11)	0.0306 (10)	0.0333 (11)	−0.0157 (9)	0.0014 (8)	−0.0106 (8)
C88	0.0282 (10)	0.0348 (11)	0.0564 (14)	−0.0157 (8)	0.0062 (9)	−0.0251 (10)
C89	0.0232 (9)	0.0371 (11)	0.0619 (14)	−0.0066 (8)	−0.0102 (9)	−0.0245 (10)
C90	0.0260 (9)	0.0281 (9)	0.0392 (11)	−0.0047 (7)	−0.0120 (8)	−0.0127 (8)
N6	0.0373 (11)	0.0650 (14)	0.0478 (13)	0.0032 (10)	−0.0042 (9)	0.0065 (11)
C93	0.0235 (10)	0.0553 (14)	0.0355 (12)	0.0000 (9)	−0.0057 (8)	−0.0077 (11)
C94	0.0267 (10)	0.0723 (17)	0.0331 (12)	−0.0060 (11)	−0.0043 (9)	−0.0024 (11)
N7	0.044 (5)	0.063 (6)	0.050 (5)	−0.022 (5)	−0.013 (4)	−0.021 (4)
N7A	0.043 (5)	0.047 (5)	0.051 (5)	−0.016 (4)	−0.017 (4)	0.020 (4)
C95	0.039 (7)	0.026 (4)	0.026 (5)	−0.018 (5)	0.003 (5)	−0.013 (4)
C95A	0.036 (7)	0.037 (6)	0.023 (6)	−0.021 (6)	0.007 (5)	−0.011 (5)
C96	0.031 (5)	0.096 (9)	0.036 (6)	0.022 (6)	0.005 (4)	−0.003 (5)
C96A	0.031 (5)	0.096 (9)	0.036 (6)	0.022 (6)	0.005 (4)	−0.003 (5)
N5	0.0647 (14)	0.0425 (11)	0.0501 (12)	−0.0274 (10)	0.0012 (10)	−0.0129 (9)
C91	0.0335 (11)	0.0296 (10)	0.0421 (12)	−0.0068 (9)	−0.0036 (9)	−0.0036 (9)
C92	0.0388 (12)	0.0487 (13)	0.0655 (16)	−0.0221 (11)	−0.0200 (11)	0.0082 (12)

### µ-Aqua-κ2O:O-di-µ-diphenylacetato-κ4O:O'-\ bis[(2,2'-bipyridine-κ2N,N')(diphenylacetato-κO)\ nickel(II)]–acetonitrile–diphenylacetic acid (1/2.5/1) (2) Geometric parameters (Å, º)

**Table:**

Ni1—O1	2.1107 (11)	C45—H45	0.9300
Ni1—O3	2.0367 (11)	C45—C46	1.388 (3)
Ni1—O5	2.0654 (11)	C46—H46	0.9300
Ni1—O9	2.0752 (11)	C46—C47	1.380 (3)
Ni1—N1	2.1154 (13)	C47—H47	0.9300
Ni1—N2	2.0672 (13)	C47—C48	1.396 (3)
Ni2—O4	2.0403 (11)	C48—H48	0.9300
Ni2—O6	2.0423 (11)	C49—C50	1.538 (2)
Ni2—O7	2.0633 (11)	C50—H50	0.9800
Ni2—O9	2.0630 (11)	C50—C51	1.523 (2)
Ni2—N3	2.0639 (13)	C50—C57	1.536 (2)
Ni2—N4	2.0921 (13)	C51—C52	1.391 (2)
O1—C49	1.2575 (19)	C51—C56	1.402 (2)
O2—C49	1.2683 (19)	C52—H52	0.9300
O3—C21	1.2546 (19)	C52—C53	1.394 (2)
O4—C21	1.2640 (19)	C53—H53	0.9300
O5—C35	1.2685 (19)	C53—C54	1.386 (3)
O6—C35	1.2497 (19)	C54—H54	0.9300
O7—C63	1.2461 (19)	C54—C55	1.385 (3)
O8—C63	1.2762 (19)	C55—H55	0.9300
O9—H9A	0.91 (3)	C55—C56	1.391 (2)
O9—H9B	0.96 (3)	C56—H56	0.9300
N1—C1	1.342 (2)	C57—C58	1.396 (2)
N1—C5	1.351 (2)	C57—C62	1.385 (3)
N2—C6	1.355 (2)	C58—H58	0.9300
N2—C10	1.338 (2)	C58—C59	1.386 (3)
N3—C11	1.336 (2)	C59—H59	0.9300
N3—C15	1.348 (2)	C59—C60	1.382 (3)
N4—C16	1.357 (2)	C60—H60	0.9300
N4—C20	1.339 (2)	C60—C61	1.381 (3)
C1—H1	0.9300	C61—H61	0.9300
C1—C2	1.391 (2)	C61—C62	1.388 (3)
C2—H2	0.9300	C62—H62	0.9300
C2—C3	1.389 (2)	C63—C64	1.529 (2)
C3—H3	0.9300	C64—H64	0.9800
C3—C4	1.386 (2)	C64—C65	1.528 (2)
C4—H4	0.9300	C64—C71	1.527 (2)
C4—C5	1.398 (2)	C65—C66	1.395 (2)
C5—C6	1.489 (2)	C65—C70	1.388 (2)
C6—C7	1.389 (2)	C66—H66	0.9300
C7—H7	0.9300	C66—C67	1.386 (3)
C7—C8	1.390 (3)	C67—H67	0.9300
C8—H8	0.9300	C67—C68	1.382 (3)
C8—C9	1.387 (3)	C68—H68	0.9300
C9—H9	0.9300	C68—C69	1.385 (3)
C9—C10	1.387 (2)	C69—H69	0.9300
C10—H10	0.9300	C69—C70	1.392 (2)
C11—H11	0.9300	C70—H70	0.9300
C11—C12	1.387 (2)	C71—C72	1.396 (2)
C12—H12	0.9300	C71—C76	1.394 (2)
C12—C13	1.386 (2)	C72—H72	0.9300
C13—H13	0.9300	C72—C73	1.391 (2)
C13—C14	1.387 (2)	C73—H73	0.9300
C14—H14	0.9300	C73—C74	1.388 (3)
C14—C15	1.394 (2)	C74—H74	0.9300
C15—C16	1.484 (2)	C74—C75	1.380 (3)
C16—C17	1.394 (2)	C75—H75	0.9300
C17—H17	0.9300	C75—C76	1.393 (3)
C17—C18	1.386 (2)	C76—H76	0.9300
C18—H18	0.9300	O10—C77	1.210 (2)
C18—C19	1.388 (2)	O11—H11A	0.8200
C19—H19	0.9300	O11—C77	1.321 (2)
C19—C20	1.385 (2)	C77—C78	1.532 (2)
C20—H20	0.9300	C78—H78	0.9800
C21—C22	1.547 (2)	C78—C79	1.528 (2)
C22—H22	0.9800	C78—C85	1.528 (2)
C22—C23	1.525 (2)	C79—C80	1.390 (3)
C22—C29	1.524 (2)	C79—C84	1.397 (3)
C23—C24	1.396 (2)	C80—H80	0.9300
C23—C28	1.399 (2)	C80—C81	1.396 (3)
C24—H24	0.9300	C81—H81	0.9300
C24—C25	1.391 (2)	C81—C82	1.390 (3)
C25—H25	0.9300	C82—H82	0.9300
C25—C26	1.393 (3)	C82—C83	1.383 (3)
C26—H26	0.9300	C83—H83	0.9300
C26—C27	1.384 (3)	C83—C84	1.393 (3)
C27—H27	0.9300	C84—H84	0.9300
C27—C28	1.392 (2)	C85—C86	1.395 (3)
C28—H28	0.9300	C85—C90	1.393 (3)
C29—C30	1.389 (3)	C86—H86	0.9300
C29—C34	1.388 (2)	C86—C87	1.392 (3)
C30—H30	0.9300	C87—H87	0.9300
C30—C31	1.391 (3)	C87—C88	1.385 (3)
C31—H31	0.9300	C88—H88	0.9300
C31—C32	1.390 (3)	C88—C89	1.382 (3)
C32—H32	0.9300	C89—H89	0.9300
C32—C33	1.376 (3)	C89—C90	1.387 (3)
C33—H33	0.9300	C90—H90	0.9300
C33—C34	1.395 (2)	N6—C93	1.133 (3)
C34—H34	0.9300	C93—C94	1.458 (3)
C35—C36	1.549 (2)	C94—H94A	0.9600
C36—H36	0.9800	C94—H94B	0.9600
C36—C37	1.521 (2)	C94—H94C	0.9600
C36—C43	1.529 (2)	N7—C95	1.184 (15)
C37—C38	1.393 (2)	N7A—C95A	1.062 (15)
C37—C42	1.397 (2)	C95—C96	1.324 (19)
C38—H38	0.9300	C95A—C96A	1.424 (17)
C38—C39	1.393 (3)	C96—H96A	0.9600
C39—H39	0.9300	C96—H96B	0.9600
C39—C40	1.385 (3)	C96—H96C	0.9600
C40—H40	0.9300	C96A—H96D	0.9600
C40—C41	1.389 (3)	C96A—H96E	0.9600
C41—H41	0.9300	C96A—H96F	0.9600
C41—C42	1.393 (2)	N5—C91	1.136 (3)
C42—H42	0.9300	C91—C92	1.452 (3)
C43—C44	1.391 (3)	C92—H92A	0.9600
C43—C48	1.393 (3)	C92—H92B	0.9600
C44—H44	0.9300	C92—H92C	0.9600
C44—C45	1.395 (3)		
			
O1—Ni1—N1	88.76 (5)	C41—C42—C37	120.72 (16)
O3—Ni1—O1	87.17 (4)	C41—C42—H42	119.6
O3—Ni1—O5	89.56 (4)	C44—C43—C36	118.50 (16)
O3—Ni1—O9	98.04 (4)	C44—C43—C48	118.20 (16)
O3—Ni1—N1	168.74 (5)	C48—C43—C36	123.25 (16)
O3—Ni1—N2	90.73 (5)	C43—C44—H44	119.4
O5—Ni1—O1	176.31 (4)	C43—C44—C45	121.14 (19)
O5—Ni1—O9	88.97 (4)	C45—C44—H44	119.4
O5—Ni1—N1	94.77 (5)	C44—C45—H45	120.0
O5—Ni1—N2	92.21 (5)	C46—C45—C44	119.92 (19)
O9—Ni1—O1	89.79 (4)	C46—C45—H45	120.0
O9—Ni1—N1	92.44 (5)	C45—C46—H46	120.2
N2—Ni1—O1	89.55 (5)	C47—C46—C45	119.63 (17)
N2—Ni1—O9	171.17 (5)	C47—C46—H46	120.2
N2—Ni1—N1	78.74 (5)	C46—C47—H47	119.8
O4—Ni2—O6	91.24 (5)	C46—C47—C48	120.32 (19)
O4—Ni2—O7	176.60 (4)	C48—C47—H47	119.8
O4—Ni2—O9	94.49 (4)	C43—C48—C47	120.80 (18)
O4—Ni2—N3	93.24 (5)	C43—C48—H48	119.6
O4—Ni2—N4	92.72 (5)	C47—C48—H48	119.6
O6—Ni2—O7	88.03 (5)	O1—C49—O2	125.58 (14)
O6—Ni2—O9	90.43 (4)	O1—C49—C50	118.18 (14)
O6—Ni2—N3	92.11 (5)	O2—C49—C50	116.19 (14)
O6—Ni2—N4	170.92 (5)	C49—C50—H50	107.7
O7—Ni2—N3	83.47 (5)	C51—C50—C49	111.94 (13)
O7—Ni2—N4	87.53 (5)	C51—C50—H50	107.7
O9—Ni2—O7	88.84 (4)	C51—C50—C57	115.15 (13)
O9—Ni2—N3	171.81 (5)	C57—C50—C49	106.33 (13)
O9—Ni2—N4	97.39 (5)	C57—C50—H50	107.7
N3—Ni2—N4	79.52 (5)	C52—C51—C50	119.45 (15)
C49—O1—Ni1	123.88 (10)	C52—C51—C56	118.11 (15)
C21—O3—Ni1	133.52 (10)	C56—C51—C50	122.44 (15)
C21—O4—Ni2	125.18 (10)	C51—C52—H52	119.4
C35—O5—Ni1	123.96 (10)	C51—C52—C53	121.11 (17)
C35—O6—Ni2	136.92 (10)	C53—C52—H52	119.4
C63—O7—Ni2	134.27 (10)	C52—C53—H53	120.0
Ni1—O9—H9A	118.5 (16)	C54—C53—C52	120.02 (17)
Ni1—O9—H9B	97.3 (16)	C54—C53—H53	120.0
Ni2—O9—Ni1	114.61 (5)	C53—C54—H54	120.1
Ni2—O9—H9A	96.8 (16)	C55—C54—C53	119.70 (16)
Ni2—O9—H9B	123.8 (16)	C55—C54—H54	120.1
H9A—O9—H9B	107 (2)	C54—C55—H55	119.9
C1—N1—Ni1	127.18 (11)	C54—C55—C56	120.25 (17)
C1—N1—C5	118.55 (14)	C56—C55—H55	119.9
C5—N1—Ni1	114.25 (10)	C51—C56—H56	119.6
C6—N2—Ni1	116.03 (11)	C55—C56—C51	120.78 (16)
C10—N2—Ni1	125.10 (11)	C55—C56—H56	119.6
C10—N2—C6	118.84 (14)	C58—C57—C50	118.56 (15)
C11—N3—Ni2	125.40 (11)	C62—C57—C50	123.18 (16)
C11—N3—C15	119.12 (13)	C62—C57—C58	118.26 (17)
C15—N3—Ni2	115.04 (10)	C57—C58—H58	119.6
C16—N4—Ni2	113.83 (10)	C59—C58—C57	120.88 (18)
C20—N4—Ni2	127.69 (11)	C59—C58—H58	119.6
C20—N4—C16	118.39 (13)	C58—C59—H59	119.9
N1—C1—H1	118.4	C60—C59—C58	120.23 (19)
N1—C1—C2	123.13 (15)	C60—C59—H59	119.9
C2—C1—H1	118.4	C59—C60—H60	120.4
C1—C2—H2	121.0	C61—C60—C59	119.29 (19)
C3—C2—C1	118.07 (15)	C61—C60—H60	120.4
C3—C2—H2	121.0	C60—C61—H61	119.7
C2—C3—H3	120.2	C60—C61—C62	120.6 (2)
C4—C3—C2	119.57 (15)	C62—C61—H61	119.7
C4—C3—H3	120.2	C57—C62—C61	120.74 (18)
C3—C4—H4	120.5	C57—C62—H62	119.6
C3—C4—C5	118.93 (15)	C61—C62—H62	119.6
C5—C4—H4	120.5	O7—C63—O8	124.10 (14)
N1—C5—C4	121.70 (15)	O7—C63—C64	117.08 (14)
N1—C5—C6	115.78 (14)	O8—C63—C64	118.80 (14)
C4—C5—C6	122.52 (14)	C63—C64—H64	107.8
N2—C6—C5	115.18 (14)	C65—C64—C63	110.54 (13)
N2—C6—C7	121.64 (15)	C65—C64—H64	107.8
C7—C6—C5	123.17 (15)	C71—C64—C63	111.36 (13)
C6—C7—H7	120.4	C71—C64—H64	107.8
C6—C7—C8	119.16 (16)	C71—C64—C65	111.45 (13)
C8—C7—H7	120.4	C66—C65—C64	117.71 (15)
C7—C8—H8	120.5	C70—C65—C64	124.37 (14)
C9—C8—C7	118.93 (16)	C70—C65—C66	117.92 (15)
C9—C8—H8	120.5	C65—C66—H66	119.3
C8—C9—H9	120.6	C67—C66—C65	121.50 (17)
C8—C9—C10	118.88 (16)	C67—C66—H66	119.3
C10—C9—H9	120.6	C66—C67—H67	120.0
N2—C10—C9	122.54 (16)	C68—C67—C66	119.99 (16)
N2—C10—H10	118.7	C68—C67—H67	120.0
C9—C10—H10	118.7	C67—C68—H68	120.4
N3—C11—H11	118.8	C67—C68—C69	119.28 (16)
N3—C11—C12	122.32 (15)	C69—C68—H68	120.4
C12—C11—H11	118.8	C68—C69—H69	119.7
C11—C12—H12	120.6	C68—C69—C70	120.57 (17)
C13—C12—C11	118.87 (16)	C70—C69—H69	119.7
C13—C12—H12	120.6	C65—C70—C69	120.72 (16)
C12—C13—H13	120.4	C65—C70—H70	119.6
C12—C13—C14	119.18 (15)	C69—C70—H70	119.6
C14—C13—H13	120.4	C72—C71—C64	121.94 (15)
C13—C14—H14	120.6	C76—C71—C64	119.37 (15)
C13—C14—C15	118.77 (15)	C76—C71—C72	118.69 (16)
C15—C14—H14	120.6	C71—C72—H72	119.7
N3—C15—C14	121.73 (15)	C73—C72—C71	120.51 (17)
N3—C15—C16	115.72 (13)	C73—C72—H72	119.7
C14—C15—C16	122.54 (14)	C72—C73—H73	120.0
N4—C16—C15	115.70 (13)	C74—C73—C72	120.07 (19)
N4—C16—C17	121.52 (14)	C74—C73—H73	120.0
C17—C16—C15	122.78 (14)	C73—C74—H74	120.0
C16—C17—H17	120.3	C75—C74—C73	119.97 (18)
C18—C17—C16	119.34 (15)	C75—C74—H74	120.0
C18—C17—H17	120.3	C74—C75—H75	119.9
C17—C18—H18	120.5	C74—C75—C76	120.10 (18)
C17—C18—C19	118.92 (15)	C76—C75—H75	119.9
C19—C18—H18	120.5	C71—C76—H76	119.7
C18—C19—H19	120.7	C75—C76—C71	120.62 (18)
C20—C19—C18	118.66 (15)	C75—C76—H76	119.7
C20—C19—H19	120.7	C77—O11—H11A	109.5
N4—C20—C19	123.08 (15)	O10—C77—O11	124.32 (16)
N4—C20—H20	118.5	O10—C77—C78	122.31 (16)
C19—C20—H20	118.5	O11—C77—C78	113.35 (15)
O3—C21—O4	127.39 (14)	C77—C78—H78	106.5
O3—C21—C22	117.61 (13)	C79—C78—C77	113.32 (14)
O4—C21—C22	115.00 (13)	C79—C78—H78	106.5
C21—C22—H22	106.5	C85—C78—C77	110.22 (14)
C23—C22—C21	109.61 (13)	C85—C78—H78	106.5
C23—C22—H22	106.5	C85—C78—C79	113.39 (14)
C29—C22—C21	112.83 (13)	C80—C79—C78	122.31 (16)
C29—C22—H22	106.5	C80—C79—C84	118.39 (17)
C29—C22—C23	114.34 (13)	C84—C79—C78	119.29 (16)
C24—C23—C22	121.23 (14)	C79—C80—H80	119.6
C24—C23—C28	118.61 (15)	C79—C80—C81	120.74 (17)
C28—C23—C22	120.16 (14)	C81—C80—H80	119.6
C23—C24—H24	119.7	C80—C81—H81	119.8
C25—C24—C23	120.58 (15)	C82—C81—C80	120.34 (18)
C25—C24—H24	119.7	C82—C81—H81	119.8
C24—C25—H25	119.9	C81—C82—H82	120.3
C24—C25—C26	120.11 (16)	C83—C82—C81	119.31 (19)
C26—C25—H25	119.9	C83—C82—H82	120.3
C25—C26—H26	120.1	C82—C83—H83	119.8
C27—C26—C25	119.86 (16)	C82—C83—C84	120.39 (19)
C27—C26—H26	120.1	C84—C83—H83	119.8
C26—C27—H27	120.0	C79—C84—H84	119.6
C26—C27—C28	120.03 (16)	C83—C84—C79	120.83 (18)
C28—C27—H27	120.0	C83—C84—H84	119.6
C23—C28—H28	119.6	C86—C85—C78	123.14 (16)
C27—C28—C23	120.79 (16)	C90—C85—C78	118.46 (16)
C27—C28—H28	119.6	C90—C85—C86	118.40 (17)
C30—C29—C22	119.11 (15)	C85—C86—H86	119.9
C34—C29—C22	122.88 (15)	C87—C86—C85	120.21 (18)
C34—C29—C30	118.00 (16)	C87—C86—H86	119.9
C29—C30—H30	119.5	C86—C87—H87	119.7
C29—C30—C31	121.05 (19)	C88—C87—C86	120.6 (2)
C31—C30—H30	119.5	C88—C87—H87	119.7
C30—C31—H31	119.9	C87—C88—H88	120.3
C32—C31—C30	120.2 (2)	C89—C88—C87	119.49 (19)
C32—C31—H31	119.9	C89—C88—H88	120.3
C31—C32—H32	120.3	C88—C89—H89	120.0
C33—C32—C31	119.38 (18)	C88—C89—C90	120.04 (19)
C33—C32—H32	120.3	C90—C89—H89	120.0
C32—C33—H33	119.9	C85—C90—H90	119.4
C32—C33—C34	120.13 (18)	C89—C90—C85	121.20 (19)
C34—C33—H33	119.9	C89—C90—H90	119.4
C29—C34—C33	121.24 (17)	N6—C93—C94	178.8 (3)
C29—C34—H34	119.4	C93—C94—H94A	109.5
C33—C34—H34	119.4	C93—C94—H94B	109.5
O5—C35—C36	115.18 (13)	C93—C94—H94C	109.5
O6—C35—O5	127.83 (14)	H94A—C94—H94B	109.5
O6—C35—C36	116.99 (13)	H94A—C94—H94C	109.5
C35—C36—H36	106.7	H94B—C94—H94C	109.5
C37—C36—C35	110.00 (13)	N7—C95—C96	176.9 (13)
C37—C36—H36	106.7	N7A—C95A—C96A	169.9 (14)
C37—C36—C43	114.12 (13)	C95—C96—H96A	109.5
C43—C36—C35	112.06 (13)	C95—C96—H96B	109.5
C43—C36—H36	106.7	C95—C96—H96C	109.5
C38—C37—C36	119.95 (15)	H96A—C96—H96B	109.5
C38—C37—C42	118.61 (16)	H96A—C96—H96C	109.5
C42—C37—C36	121.44 (14)	H96B—C96—H96C	109.5
C37—C38—H38	119.6	C95A—C96A—H96D	109.5
C37—C38—C39	120.81 (17)	C95A—C96A—H96E	109.5
C39—C38—H38	119.6	C95A—C96A—H96F	109.5
C38—C39—H39	120.0	H96D—C96A—H96E	109.5
C40—C39—C38	119.95 (17)	H96D—C96A—H96F	109.5
C40—C39—H39	120.0	H96E—C96A—H96F	109.5
C39—C40—H40	120.0	N5—C91—C92	179.1 (3)
C39—C40—C41	120.03 (17)	C91—C92—H92A	109.5
C41—C40—H40	120.0	C91—C92—H92B	109.5
C40—C41—H41	120.1	C91—C92—H92C	109.5
C40—C41—C42	119.85 (18)	H92A—C92—H92B	109.5
C42—C41—H41	120.1	H92A—C92—H92C	109.5
C37—C42—H42	119.6	H92B—C92—H92C	109.5
			
Ni1—O1—C49—O2	−9.0 (2)	C32—C33—C34—C29	0.6 (3)
Ni1—O1—C49—C50	168.31 (10)	C34—C29—C30—C31	−0.6 (3)
Ni1—O3—C21—O4	−11.4 (2)	C35—C36—C37—C38	−136.86 (15)
Ni1—O3—C21—C22	168.64 (10)	C35—C36—C37—C42	43.5 (2)
Ni1—O5—C35—O6	−17.7 (2)	C35—C36—C43—C44	79.46 (19)
Ni1—O5—C35—C36	162.19 (10)	C35—C36—C43—C48	−97.84 (18)
Ni1—N1—C1—C2	−178.37 (12)	C36—C37—C38—C39	−178.49 (15)
Ni1—N1—C5—C4	−179.59 (12)	C36—C37—C42—C41	178.79 (15)
Ni1—N1—C5—C6	0.31 (17)	C36—C43—C44—C45	−177.24 (16)
Ni1—N2—C6—C5	1.56 (17)	C36—C43—C48—C47	177.35 (16)
Ni1—N2—C6—C7	−177.95 (12)	C37—C36—C43—C44	−154.68 (15)
Ni1—N2—C10—C9	177.38 (12)	C37—C36—C43—C48	28.0 (2)
Ni2—O4—C21—O3	−30.4 (2)	C37—C38—C39—C40	−0.2 (3)
Ni2—O4—C21—C22	149.50 (10)	C38—C37—C42—C41	−0.8 (2)
Ni2—O6—C35—O5	−19.0 (3)	C38—C39—C40—C41	−1.1 (3)
Ni2—O6—C35—C36	161.12 (11)	C39—C40—C41—C42	1.4 (3)
Ni2—O7—C63—O8	−6.9 (2)	C40—C41—C42—C37	−0.4 (3)
Ni2—O7—C63—C64	171.48 (10)	C42—C37—C38—C39	1.1 (2)
Ni2—N3—C11—C12	172.60 (12)	C43—C36—C37—C38	96.21 (18)
Ni2—N3—C15—C14	−174.00 (12)	C43—C36—C37—C42	−83.38 (18)
Ni2—N3—C15—C16	4.83 (17)	C43—C44—C45—C46	−0.3 (3)
Ni2—N4—C16—C15	−0.71 (16)	C44—C43—C48—C47	0.0 (3)
Ni2—N4—C16—C17	178.80 (11)	C44—C45—C46—C47	0.1 (3)
Ni2—N4—C20—C19	−175.35 (12)	C45—C46—C47—C48	0.1 (3)
O1—C49—C50—C51	123.84 (15)	C46—C47—C48—C43	−0.2 (3)
O1—C49—C50—C57	−109.61 (16)	C48—C43—C44—C45	0.2 (3)
O2—C49—C50—C51	−58.63 (18)	C49—C50—C51—C52	−117.76 (16)
O2—C49—C50—C57	67.92 (17)	C49—C50—C51—C56	62.9 (2)
O3—C21—C22—C23	−116.76 (15)	C49—C50—C57—C58	50.4 (2)
O3—C21—C22—C29	11.9 (2)	C49—C50—C57—C62	−129.54 (18)
O4—C21—C22—C23	63.30 (17)	C50—C51—C52—C53	−178.72 (15)
O4—C21—C22—C29	−168.04 (13)	C50—C51—C56—C55	177.84 (15)
O5—C35—C36—C37	63.75 (18)	C50—C57—C58—C59	−177.98 (17)
O5—C35—C36—C43	−168.18 (14)	C50—C57—C62—C61	177.9 (2)
O6—C35—C36—C37	−116.34 (15)	C51—C50—C57—C58	174.98 (15)
O6—C35—C36—C43	11.7 (2)	C51—C50—C57—C62	−5.0 (2)
O7—C63—C64—C65	−80.18 (17)	C51—C52—C53—C54	0.7 (3)
O7—C63—C64—C71	44.29 (19)	C52—C51—C56—C55	−1.5 (2)
O8—C63—C64—C65	98.32 (16)	C52—C53—C54—C55	−1.2 (3)
O8—C63—C64—C71	−137.21 (15)	C53—C54—C55—C56	0.3 (3)
N1—C1—C2—C3	−1.4 (3)	C54—C55—C56—C51	1.0 (3)
N1—C5—C6—N2	−1.2 (2)	C56—C51—C52—C53	0.6 (2)
N1—C5—C6—C7	178.27 (15)	C57—C50—C51—C52	120.64 (16)
N2—C6—C7—C8	0.0 (2)	C57—C50—C51—C56	−58.7 (2)
N3—C11—C12—C13	0.1 (3)	C57—C58—C59—C60	−0.3 (3)
N3—C15—C16—N4	−2.7 (2)	C58—C57—C62—C61	−2.0 (3)
N3—C15—C16—C17	177.77 (14)	C58—C59—C60—C61	−1.3 (4)
N4—C16—C17—C18	−3.4 (2)	C59—C60—C61—C62	1.2 (4)
C1—N1—C5—C4	2.0 (2)	C60—C61—C62—C57	0.5 (4)
C1—N1—C5—C6	−178.09 (13)	C62—C57—C58—C59	2.0 (3)
C1—C2—C3—C4	1.3 (3)	C63—C64—C65—C66	−172.01 (14)
C2—C3—C4—C5	0.4 (2)	C63—C64—C65—C70	8.8 (2)
C3—C4—C5—N1	−2.1 (2)	C63—C64—C71—C72	−64.78 (19)
C3—C4—C5—C6	177.99 (15)	C63—C64—C71—C76	114.66 (16)
C4—C5—C6—N2	178.67 (14)	C64—C65—C66—C67	−177.99 (16)
C4—C5—C6—C7	−1.8 (2)	C64—C65—C70—C69	178.10 (16)
C5—N1—C1—C2	−0.2 (2)	C64—C71—C72—C73	177.64 (15)
C5—C6—C7—C8	−179.47 (15)	C64—C71—C76—C75	−178.55 (16)
C6—N2—C10—C9	−1.0 (2)	C65—C64—C71—C72	59.17 (19)
C6—C7—C8—C9	−0.1 (3)	C65—C64—C71—C76	−121.40 (16)
C7—C8—C9—C10	−0.3 (3)	C65—C66—C67—C68	−0.1 (3)
C8—C9—C10—N2	0.8 (3)	C66—C65—C70—C69	−1.1 (3)
C10—N2—C6—C5	−179.97 (14)	C66—C67—C68—C69	−1.1 (3)
C10—N2—C6—C7	0.5 (2)	C67—C68—C69—C70	1.3 (3)
C11—N3—C15—C14	−1.3 (2)	C68—C69—C70—C65	−0.2 (3)
C11—N3—C15—C16	177.58 (13)	C70—C65—C66—C67	1.2 (3)
C11—C12—C13—C14	−0.4 (2)	C71—C64—C65—C66	63.58 (19)
C12—C13—C14—C15	−0.2 (2)	C71—C64—C65—C70	−115.60 (18)
C13—C14—C15—N3	1.0 (2)	C71—C72—C73—C74	1.2 (3)
C13—C14—C15—C16	−177.73 (15)	C72—C71—C76—C75	0.9 (3)
C14—C15—C16—N4	176.09 (14)	C72—C73—C74—C75	0.4 (3)
C14—C15—C16—C17	−3.4 (2)	C73—C74—C75—C76	−1.2 (3)
C15—N3—C11—C12	0.7 (2)	C74—C75—C76—C71	0.6 (3)
C15—C16—C17—C18	176.08 (15)	C76—C71—C72—C73	−1.8 (2)
C16—N4—C20—C19	1.0 (2)	O10—C77—C78—C79	144.02 (18)
C16—C17—C18—C19	1.8 (2)	O10—C77—C78—C85	−87.7 (2)
C17—C18—C19—C20	1.0 (2)	O11—C77—C78—C79	−37.5 (2)
C18—C19—C20—N4	−2.5 (3)	O11—C77—C78—C85	90.73 (18)
C20—N4—C16—C15	−177.53 (13)	C77—C78—C79—C80	85.1 (2)
C20—N4—C16—C17	2.0 (2)	C77—C78—C79—C84	−93.60 (19)
C21—C22—C23—C24	42.37 (19)	C77—C78—C85—C86	−37.8 (2)
C21—C22—C23—C28	−136.44 (14)	C77—C78—C85—C90	142.80 (16)
C21—C22—C29—C30	89.29 (19)	C78—C79—C80—C81	−178.50 (16)
C21—C22—C29—C34	−89.59 (19)	C78—C79—C84—C83	178.14 (18)
C22—C23—C24—C25	179.96 (15)	C78—C85—C86—C87	−179.00 (17)
C22—C23—C28—C27	178.87 (14)	C78—C85—C90—C89	178.42 (17)
C22—C29—C30—C31	−179.56 (19)	C79—C78—C85—C86	90.4 (2)
C22—C29—C34—C33	178.85 (16)	C79—C78—C85—C90	−88.97 (19)
C23—C22—C29—C30	−144.56 (16)	C79—C80—C81—C82	0.2 (3)
C23—C22—C29—C34	36.6 (2)	C80—C79—C84—C83	−0.6 (3)
C23—C24—C25—C26	1.5 (2)	C80—C81—C82—C83	−0.2 (3)
C24—C23—C28—C27	0.0 (2)	C81—C82—C83—C84	−0.2 (3)
C24—C25—C26—C27	−0.6 (2)	C82—C83—C84—C79	0.6 (3)
C25—C26—C27—C28	−0.6 (2)	C84—C79—C80—C81	0.2 (3)
C26—C27—C28—C23	0.9 (2)	C85—C78—C79—C80	−41.5 (2)
C28—C23—C24—C25	−1.2 (2)	C85—C78—C79—C84	139.79 (17)
C29—C22—C23—C24	−85.45 (18)	C85—C86—C87—C88	0.6 (3)
C29—C22—C23—C28	95.74 (17)	C86—C85—C90—C89	−1.0 (3)
C29—C30—C31—C32	0.7 (4)	C86—C87—C88—C89	−0.9 (3)
C30—C29—C34—C33	0.0 (3)	C87—C88—C89—C90	0.3 (3)
C30—C31—C32—C33	−0.1 (4)	C88—C89—C90—C85	0.7 (3)
C31—C32—C33—C34	−0.6 (3)	C90—C85—C86—C87	0.4 (3)

### µ-Aqua-κ2O:O-di-µ-diphenylacetato-κ4O:O'-\ bis[(2,2'-bipyridine-κ2N,N')(diphenylacetato-κO)\ nickel(II)]–acetonitrile–diphenylacetic acid (1/2.5/1) (2) Hydrogen-bond geometry (Å, º)

**Table:**

*D*—H···*A*	*D*—H	H···*A*	*D*···*A*	*D*—H···*A*
O9—H9*A*···O8	0.91 (3)	1.74 (3)	2.6461 (16)	173 (2)
O9—H9*B*···O2	0.96 (3)	1.53 (3)	2.4777 (15)	169 (3)
O11—H11*A*···O8	0.82	1.76	2.5599 (16)	164
C4—H4···O5^i^	0.93	2.48	3.3253 (19)	152
C17—H17···O4^ii^	0.93	2.48	3.3493 (19)	155
C10—H10···O3	0.93	2.46	3.015 (2)	118
C11—H11···O6	0.93	2.52	3.0646 (19)	118
C20—H20···O2	0.93	2.32	3.2283 (19)	167
C94—H94*B*···O2	0.96	2.55	3.295 (2)	135

Symmetry codes: (i) −*x*+1, −*y*+1, −*z*+2; (ii) −*x*+1, −*y*+2, −*z*+1.
